# Reporting of social determinants of health in randomized controlled trials conducted in the pediatric intensive care unit

**DOI:** 10.3389/fped.2024.1329648

**Published:** 2024-02-01

**Authors:** Emma Huang, Lisa Albrecht, Katie O’Hearn, Naisha Nicolas, Jennifer Armstrong, Maya Weinberg, Kusum Menon

**Affiliations:** ^1^ Faculty of Medicine, University of Ottawa, Ottawa, ON, Canada; ^2^ Children’s Hospital of Eastern Ontario Research Institute, Ottawa, ON, Canada; ^3^ Faculty of Science, University of Ottawa, Ottawa, ON, Canada; ^4^ Department of Pediatrics, Children’s Hospital of Eastern Ontario and University of Ottawa, Ottawa, ON, Canada

**Keywords:** social determinants of health, randomized controlled trial, pediatric intensive care unit, critical illness, children

## Abstract

**Introduction:**

The influence of social determinants of health (SDOH) on access to care and outcomes for critically ill children remains an understudied area with a paucity of high-quality data. Recent publications have highlighted the importance of incorporating SDOH considerations into research but the frequency with which this occurs in pediatric intensive care unit (PICU) research is unclear. Our objective was to determine the frequency and categories of SDOH variables reported and how these variables were defined in published PICU randomized controlled trials (RCTs).

**Methods:**

We searched Medline, Embase, Lilacs, and Central from inception to Dec 2022. Inclusion criteria were randomized controlled trials of any intervention on children or their families in a PICU. Data related to study demographics and nine WHO SDOH categories were extracted, and descriptive statistics and qualitative data generated.

**Results:**

586 unique RCTs were included. Studies had a median sample size of 60 patients (IQR 40-106) with 73.0% of studies including ≤100 patients and 41.1% including ≤50 patients. A total of 181 (181/586, 30.9%) studies reported ≥1 SDOH variable of which 163 (163/586, 27.8%) reported them by randomization group. The most frequently reported categories were food insecurity (100/586, 17.1%) and social inclusion and non-discrimination (73/586, 12.5%). Twenty-five of 57 studies (43.9%) investigating feeding or nutrition and 11 of 82 (13.4%) assessing mechanical ventilation reported baseline nutritional assessments. Forty-one studies investigated interventions in children with asthma or bronchiolitis of which six reported on smoking in the home (6/41, 14.6%).

**Discussion:**

Reporting of relevant SDOH variables occurs infrequently in PICU RCTs. In addition, when available, categorizations and definitions of SDOH vary considerably between studies. Standardization of SDOH variable collection along with consistent minimal reporting requirements for PICU RCT publications is needed.

## 1 Introduction

The study of how social determinants of health (SDOH) influence access to care and outcomes for critically ill children is a developing area of research, with a paucity of high-quality studies. There is emerging evidence that SDOH may affect health outcomes (1–6) and recent publications have highlighted the importance of incorporating SDOH considerations into research conducted in pediatric intensive care units (PICUs) (1, 7, 8). Randomized controlled trials (RCTs) are often considered the gold standard of clinical evidence (9, 10) which underscores the importance of including of SDOH variables in their design, data collection and analysis.

Inclusion of SDOH variables in RCTs is important for several reasons. Firstly, if RCTs do not incorporate SDOH in the baseline demographics of the study population or in the subsequent analysis, this may lead to confounding of study results. This is particularly important in PICU RCTs, which typically have small sample sizes rendering them more susceptible to the impacts of unbalanced study arms. Secondly, if SDOH variables are not consistently reported, it becomes difficult to determine the generalizability of study results. Detailed information on SDOH variables allows one to determine (1) whether the recruited patients represent those most affected by the disease, (2) whether the recruited patients are similar to the population from which they are drawn and (3) whether the study population is similar to that served by clinicians reading the results (11, 12).

Finally, results from small sample size RCTs may sometimes be strengthened by combining studies in systematic reviews and meta-analyses. However, lack of reporting of SDOH and variability in the definition of reported SDOH variables may limit the ability to combine studies (7). Therefore, the objective of this study was to determine the frequency and categories of SDOH variables reported and how these variables were defined in published PICU RCTs.

## 2 Methods

### 2.1 Data source and search strategy

The database of RCTs used for this study was initially developed as described in a previously published scoping review (13). The database was updated by searching MEDLINE, EMBASE, LILACS and CENTRAL up to December 31st, 2022, as per the original search strategy. The articles retrieved from the search were reviewed and selected as per the previously described methodology (13). Inclusion criteria for studies were: (1) randomized controlled trial; and (2) any intervention on children or their families in a pediatric intensive care unit. Studies meeting the following criteria were excluded: (1) involving only preterm infants or infants in a neonatal intensive care unit; (2) individual patient crossover trials; (3) published only as abstracts or study protocols; (4) sub study or secondary analysis of an included RCT. A unit was considered a PICU or critical care unit if defined as such by the authors and if it had the ability to provide mechanical ventilation. We included trials in all languages and used the most recent publication for trials reporting results in multiple papers. This study included all articles in the updated database (i.e., from 1986 to 2022).

### 2.2 Generation of SDOH variables

Data collection was based on the WHO categories of SDOH (14). We included nine SDOH categories provided by the WHO: income and social protection; early childhood development; (parental) unemployment and job security; food insecurity; structural conflict; education; social inclusion and non-discrimination; housing, basic amenities, and the environment; and access to affordable health services (14). We excluded working life conditions as a category as it was not considered to be directly relevant to the health of children. Country income level was defined as per the World Bank classification (15) based on the publication year of the article. The category of early childhood development is influenced by numerous factors that overlap with other categories such as food insecurity and housing, basic amenities and the environment (16). Therefore, only those variables that were unique to early childhood development were included under this category (caregiver marital status, parental support systems and household composition).

We searched Medline and PubMed as of November 2021 for articles reporting specific variables within each of the nine relevant WHO SDOH categories. We used variables derived from these articles to create the data extraction form and augmented it with variables from a previous study (7) to ensure inclusion of a broad range of possible SDOH variables.

### 2.3 Data extraction

The data extraction form was piloted by two reviewers using 25 articles from the previously published scoping review (13). Once refined, two reviewers independently extracted data from all RCTs in duplicate and conflicts were resolved via consensus. For papers published in a language other than English, reviewers who read the respective language fluently were recruited and trained to complete the data extraction form. If only one fluent reviewer was available to extract an article in a particular language, the second reviewer completed the data extraction form using web-based translation software (17).

### 2.4 Data collection and analysis

Key data extraction components from each study included reported study and participant demographics, information on SDOH variables and study primary intervention(s). The reporting of SDOH variables per study cohort or randomization arm was also recorded. Study characteristics were reported descriptively using counts with percentages or measures of central tendency and variance and represented via text, tables and figures.

## 3 Results

**Figure 1:**
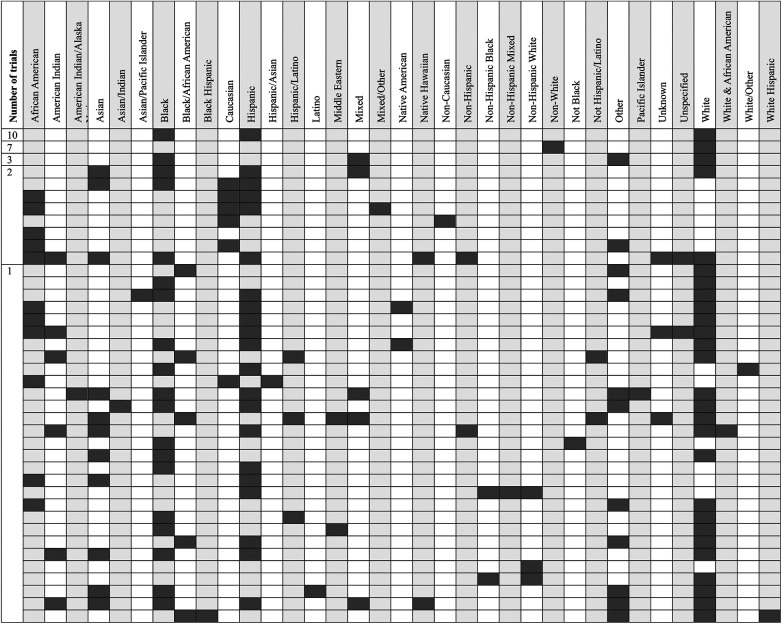
Number of randomized controlled trials (RCTs) and percentage of trials reporting social determinants of health over time. Years prior to 1996 had ≤5 RCTs and therefore were excluded from the graph.

The demographics of the 586 included RCTs are shown in Table 1 and the references for these RCTs are provided in the Supplementary Material. Four hundred and fifty-three studies were conducted in high-income (HIC) or upper-middle income countries (UMIC) (453/586, 77.3%), 132 (132/586, 22.5%) in lower-middle income countries (LMIC) and one study in both HIC and LMIC settings. No studies were conducted in low-income countries (LIC). Included studies had a median sample size of 60 patients (IQR 40–106) with 73.0% of studies (428/586) including ≤100 patients and 41.1% (241/586) including ≤50 patients. The number of RCTs per year along with the number reporting any SDOH variable for either the whole study cohort or by study arm is shown in Figure 1. A total of 181 (193/586, 30.9%) studies reported ≥1 SDOH variable of which 163 (163/586, 27.8%) reported them by randomization group. The SDOH categories reported are shown in Table 2. Most studies (156/586, 26.6%) reported on only one of the predetermined WHO categories. Of the studies that investigated at least one SDOH, the most frequently reported category was food insecurity (100/586, 17.1%), followed by social inclusion and non-discrimination (73/586, 12.5%). The interventions studied in included trials are listed in Table 3. The specific references pertaining to the results below are summarized in the Supplementary Materials File S2.

**Table 1:** Demographics of included studies.

Demographics	*N* (%)
Participating countries	
USA	150 (25.6)
India	69 (11.8)
China	52 (8.9)
Brazil	34 (5.8)
Canada	33 (5.6)
Egypt, United Kingdom	27 each (4.6)
Australia, Iran, Turkey	24 each (4.1)
Netherlands	19 (3.2)
Italy	16 (2.7)
Spain	15 (2.6)
Germany	14 (2.4)
Thailand	13 (2.2)
France	11 (1.9)
Chile, New Zealand	8 (1.4) each
Belgium, Japan	7 (1.2) each
Argentina	6 (1.0)
Greece, Israel, South Korea, Switzerland	5 (0.9) each
Austria, Russia, South Africa, Vietnam	4 (0.7) each
Finland, Indonesia, Saudi Arabia	3 (0.5) each
Croatia, Cuba, Pakistan, Poland	2 (0.3) each
Bangladesh, Columbia, Malaysia, Mexico, Norway, Peru, Philippines, Portugal, Slovakia, Sweden, Taiwan, Tunisia, Ukraine	1 (0.2) each
Publication language	
English	566 (96.6)
Chinese	11 (1.9)
Portuguese	3 (0.5)
Spanish	2 (0.3)
German	1 (0.2)
Italian	1 (0.2)
Russian	1 (0.2)
Turkish	1 (0.2)
Patient ages	
Neonates (<1 month)	145 (24.7)
Infants (1 month–1 year)	332 (56.7)
Preschool (1 year–5 years)	341 (58.2)
School-age (5 years–12 years)	280 (47.8)
Adolescents (12 years–18 years)	208 (35.5)
All ages	163 (27.8)

**Table 2:** Social determinants of health categories and variables reported.

Social determinant of health category	Potentially related variables reported	Total number of studies reporting	
		By trial arm, *n* (%)	By overall cohort, *n* (%)
Food insecurity	Height, weight, other measures, nutritional status, nutrient levels	97 (16.6)	3 (0.5)
Social inclusion and non-discrimination	Patient race/ethnicity, caregiver race/ethnicity	68 (11.6)	5 (0.9)
Income and social protection	Country income level, patient/family income level, patient/family socioeconomic status, patient/family poverty	4 (0.7)	6 (10)
Affordable healthcare access	Travel time to healthcare, health insurance status, PICU in nearest hospital, immunization status	3 (0.5)	7 (1.2)
Housing and basic amenities	House environment, neighbourhood environment	9 (1.5)	2 (0.3)
Early childhood development	Caregiver social supports, marital status of caregivers, household size	5 (0.9)	3 (0.5)
Education	Maternal and paternal education	4 (0.7)	2 (0.3)
Unemployment and job security	Caregiver employment status	1 (0.2)	1 (0.2)
Structural conflict	Child protective services	0 (0.0)	2 (0.3)

**Table 3:** Interventions studied in participating trials.

Type of intervention	*N*
Drug	236
Ventilation	82
Anesthesia/analgesia	58
Nutrition	57
Fluid management	33
Infection control	21
Imaging	17
Alternative therapies	11
Hypothermia	11
Transfusion	10
Physical therapy/exercise/rehabilitation	7
Monitoring	7
Parental support	6
Plasma exchange/filtration	5
Practise guidelines	5
Renal replacement	3
Central venous catheter procedures	2
Ischemia and reperfusion	2
Suctioning practices	2
Other^a^	10

^a^ Other interventions with one study per category included abuse evaluation tool, cardiac resynchronization, corneal protection, fast tracking cardiac surgery, hemodynamic optimization, nitric oxide, helium, resident shifts, temperature thresholds, glycemic control, and genome sequencing.

### 3.1 Food insecurity

A total of 17.1% (100/586) studies reported a potential food insecurity factor (other than admission weight, height, and/or BMI alone). Ninety-seven of which reported by randomization group and three for the whole study cohort. Studies reported 12 different measures of nutritional status with variable definitions of these measures. Reporting of direct measures of height and weight also varied and included height and weight for age, height and weight for age *z*-scores, weight for height, weight for height *z*-scores, body surface area, and body mass index *z*-score. Twelve studies provided laboratory measures and included mineral, albumin and cholesterol levels. Twenty-five of 57 studies (43.9%) investigating feeding or nutrition reported baseline nutritional assessments. Of the remaining 32 studies, only five had a sample size >100 subjects. Eleven of 82 studies assessing mechanical ventilation (13.4%) reported baseline nutritional assessments and of the remaining 71 trials, 48 had a sample size <100 (67.6%).

### 3.2 Social inclusion and non-discrimination

**Figure 2:**
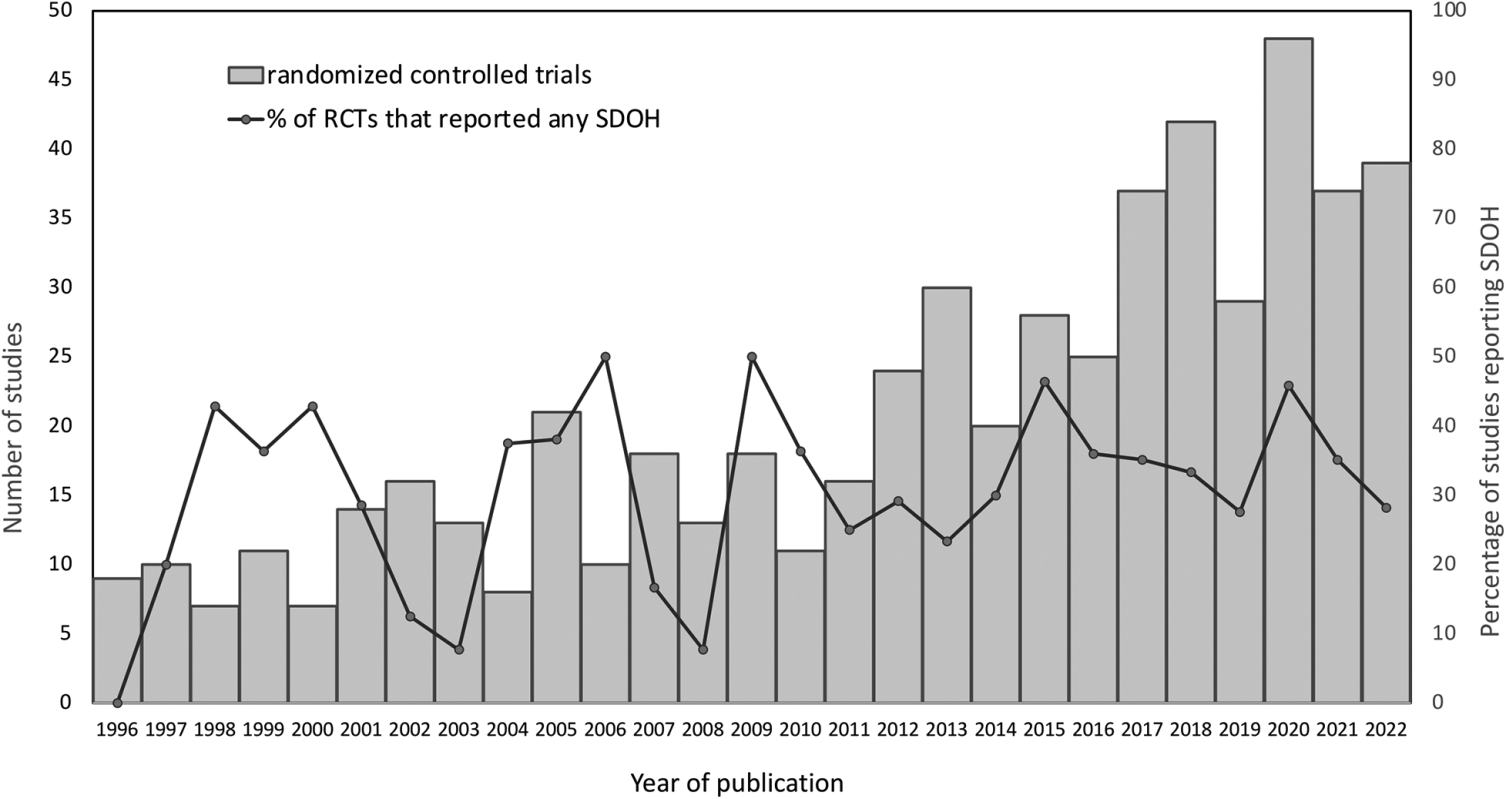
Categories of race and ethnicity reported in included studies.

Most RCTs (501/586, 85.5%) reported on the sex of participating patients; however, no study reported on non-binary gender variables. Race/ethnicity of the patient and/or caregiver was reported in 73 RCTs (73/586, 12.5%). Most studies that reported on race/ethnicity were conducted in the United States (61/73, 83.6%) and the 61 studies used 40 different categorization combinations (Figure 2). Sixteen studies (16/73, 21.9%) described the method by which race/ethnicity was determined and included family member self-report (7/16), medical records (5/16), categorization by study team (3/16) and categorization by study team in consultation with family (1/16). No study commented on whether the race/ethnicity of the study population reflected that of the referral population of the participating site.

### 3.3 Access to affordable health service*s*

An aspect of affordable health services was investigated by 1.7% of studies (10/586). Four studies (4/586, 0.7%) reported on patient travel distance to health services, one of which reported distance by study arm. One study commented on the presence of a PICU in the nearest hospital, one study reported on patient health insurance status and one study on patient immunization status. Five studies reported on urban vs. rural populations: four for the whole study population and one by study arm.

### 3.4 Income and social protection

Socioeconomic status (SES) of the patient/family was mentioned in 10 (10/586, 1.7%) of the included publications. Six studies described the SES of the overall study cohort and four studies reported on the percentage of patients from families from low SES backgrounds in each study arm. One study provided a basic description of SES by the immediate catchment area and another by the recruited patient cohort, but neither described further variables by randomization arm. None of the 10 studies described how SES was defined or determined.

### 3.5 Early childhood development

The age distribution of children in included trials is shown in Table 1. Four hundred studies (569/586, 97.1%) included neonates, infants and/or preschool aged children. Six studies reported on parent's marital status (6/586, 1.0%), four by randomization arm and two for the overall cohort. Two studies (2/586, 0.3%) reported on household composition, one by randomization arm and one for the overall study cohort.

### 3.6 Housing, basic amenities, and the environment

Eleven studies (11/586, 1.9%) reported on the patient's neighborhood environment, of which eight (8/11, 72.7%) reported on a patient's exposure to caregiver smoking, seven studies per study arm and one for the whole cohort. Two studies described the overall patient cohort as living at high altitude and one study reported on organophosphate poisoning frequency per study arm. Sixteen studies investigated interventions in children with severe asthma of which three studies reported on smoking in the home. Similarly, 25 studies assessed interventions in children with bronchiolitis, three of which reported on patient exposure to smoke in the home. Thirty-one of the 41 studies on asthma and bronchiolitis enrolled ≤100 patients and 24 enrolled ≤50 patients.

### 3.7 Education

Six studies (6/586, 1.0%) reported the education and/or schooling level of the caregiver, four by study arm and two for the whole cohort. No studies investigated the education and/or schooling level of the child.

### 3.8 Unemployment and job security

Two studies (2/586, 0.3%) conducted among patient caregivers reported on their employment status, one trial reported by study arm and the other for the overall cohort. No trial commented on patient employment status although 372 studies (372/586, 63.5%) included adolescents in their study population and 15 studies explicitly stated that they included patients above 18 years of age.

### 3.9 Structural conflict

Two studies reported on enrolled patients in the overall cohort being under the care of Child Protective Services but did not describe which study arm they belonged to. No trial reported on the presence of armed conflict in the participating regions of the study.

## 4 Discussion

Our study found that just under a third of randomized controlled trials conducted in PICU since 1986 reported baseline patient data for one or more SDOH categories and that most studies reported only one SDOH variable. Important variables known to affect children's health such as smoking in the household (18–22), maternal education level (22) and socioeconomic status (23–26) were seldom reported. The most frequently reported SDOH categories were food insecurity (with nutritional status as a potential surrogate marker), and social inclusion and non-discrimination (race/ethnicity). The definitions for collected variables were infrequently provided and showed considerable variation when reported. Importantly, almost three-quarters of included studies had sample sizes ≤100 patients.

The potential issue raised by small sample sizes is highlighted by the data reported for smoking in the household. The association of incidence and severity of respiratory diseases in children with second hand smoke is well documented (18–21). Despite this, only 3/16 studies assessing interventions for asthma and 3/25 studies assessing interventions for bronchiolitis reported on smoking in the house. Given that more than 80% of the asthma and bronchiolitis studies had sample sizes ≤100 (27), lack of control for smoking as a confounder may have significantly biased the results (28, 29).

Similarly, time to reach the desired caloric goal for patients in PICU has been linked to pre-existing malnutrition (30). However, almost half the trials investigating a feeding or nutrition intervention did not report baseline nutritional status and 80% of these trials had a sample size ≤100 subjects. Nutritional status on admission to PICU has also been shown to be an independent predictor of duration of mechanical ventilation (31, 32) and yet only 11 of 82 RCTs assessing mechanical ventilation as an intervention reported baseline nutritional status. It is possible that malnutrition, especially in high income settings, may reflect chronic illness rather than food insecurity. However, it is still important to report nutritional status along with the prevalence of chronic disease at baseline to allow meaningful comparisons between randomization groups and incorporation of nutritional status as a potential confounder of the relationship between the intervention and studied outcome. Finally, nutritional status in included studies was measured using a wide variety of categories and definitions making comparisons between studies of similar interventions difficult.

We also found significant variation in the categories and definitions of race and ethnicity data reported which is consistent with the findings of others (33). Our study also found that trials conducted outside of the US rarely reported race/ethnicity data and US based trials reported race/ethnicity data less than 50% of the time despite the existence of federal government mandates and standards since 2002 (34). Furthermore, the US government suggests a minimum of five categories for race (American Indian/Alaska Native, Asian, Black/African American, Native Hawaiian/Other Pacific Islander, and White) (34), but only four US based trials used these categories. However, despite the challenges in collecting and categorizing race/ethnicity data, researchers have a responsibility to conduct studies that strive to improve understanding of health disparities, acknowledge structural mediators such as racism and provide opportunities to advance health equity (1, 35–40). Numerous other SDOH factors may confound the apparent relationship between race/ethnicity and outcomes (36–38, 41, 42) and thus result in treatment inequities (43). Therefore, rigorous analytic models that account for the complex relationships between certain SDOH variables (such as race/ethnicity) and health outcomes are required even in RCTs (36, 37).

The variation in definitions used for nutrition and race/ethnicity categories highlights the need for standardization of how these and other variables are defined and reported. An international collaboration, with representation from different settings, to develop consensus definitions and a minimum set of SDOH variables that should be reported for all PICU trials would be ideal. International working groups with diverse membership have successfully collaborated on unified definitions for pediatric acute respiratory distress syndrome (44, 45) and pediatric sepsis (46, 47) demonstrating that consensus building endeavors in PICU are both possible and effective.

Although the trials in our study were conducted in 49 different countries each of which was classified as a LMIC, UMIC or HIC setting, the SES of the actual recruited study population was only delineated in two studies. This is an important distinction as high-income countries may have lower resourced settings within them, and lower income countries may also encompass well-resourced settings (48). Therefore, to determine the generalizability of the results of a given trial, it is important to have detailed patient level baseline SES in addition to the SES of the hospital catchment area population. This also applies to other SDOH variables such as structural conflict and caregiver unemployment which although might not have direct applicability to the interventions or outcomes being investigated, may still allow readers to assess the generalizability of the results (49). Of note, the search strategy for the database used for this study required that included RCTs needed to have been conducted in a PICU. Therefore, some studies conducted on critically ill children in LICs where PICU resources are limited may have been excluded (50).

Finally, certain variables such as immunization status were rarely reported. While it could be argued that immunization status in some patients results from family preferences, there are also several studies suggesting an association between immunization rates and education levels, family income and access to care (51–53). In addition to be a potential surrogate for other social determinants of health, immunization status could be a confounder for illness severity in certain diseases and as such be important to report on in RCTs.

### 4.1 Limitations

There were several limitations to our study. This was a retrospective study of previously published articles. Therefore, we could not determine whether certain reported variables were truly represented SDOH or were collected for other reasons. Despite this, the low overall rate of reporting for SDOH was significant. Another limitation was that the RCTs reviewed in our study only included those conducted in a PICU and therefore did not include any LICs. This would be an important area for future study.

## 5 Conclusion

Our study highlights areas for improvement in the collection and reporting of SDOH variables. A concerted global effort to develop a minimum SDOH variable dataset along with standardized definitions and methods for commonly collected SDOH variables such as nutritional status and race/ethnicity would be especially valuable.

## Data Availability

The original contributions presented in the study are included in the article/**Supplementary Material**, further inquiries can be directed to the corresponding author.
