# Supplementary material for: Reporting of social determinants of health in randomized controlled trials conducted in the pediatric intensive care unit
Source: Front Pediatr. 2024 Feb 1;12:1329648. doi: 10.3389/fped.2024.1329648 (PMC10867174; doi:10.3389/fped.2024.1329648)
Supplement: Supplementary file 1 [file Table1.docx]

**Supplementary file 1: References for included RCTs**

**Randomized Controlled Trials Included**

1. Simpson SA, Zaccagni H, Bichell DP, Christian KG, Mettler BA, Donahue BS, et al. Acetaminophen attenuates lipid peroxidation in children undergoing cardiopulmonary bypass. Pediatric Critical Care Medicine. 2014;15(6):503–10.

2. Rodrigues GO, Medeiros RF, Rodrigues SC, Boll LFC, Irigoyen MC, Goldmeier S. Choice of tube extremity for emission of the lowest radiation dose in pediatric patients. IJC Heart and Vasculature. 2018 Dec 1;21:64–8.

3. Jain P, Rameshkumar R, Satheesh P, Mahadevan S. Early Goal-Directed Therapy With and Without Intermittent Superior Vena Cava Oxygen Saturation Monitoring in Pediatric Septic Shock: A Randomized Controlled Trial. Indian Pediatr. 2021;58:1124–30.

4. Cambonie G, Milési C, Fournier-Favre S, Counil F, Picaud JC, Matecki S. Clinical Effects of Heliox Administration for Acute Bronchiolitis in Young Infants*. Chest [Internet]. 2006;129(3):676–82. Available from: http://journal.publications.chestnet.org/

5. Milési C, Matecki S, Jaber S, Mura T, Jacquot A, Pidoux O, et al. 6 cmH2O continuous positive airway pressure versus conventional oxygen therapy in severe viral bronchiolitis: A randomized trial. Pediatr Pulmonol. 2013 Jan 1;48(1):45–51.

6. Chisti MJ, Salam MA, Smith JH, Ahmed T, Pietroni MAC, Shahunja KM, et al. Bubble continuous positive airway pressure for children with severe pneumonia and hypoxaemia in Bangladesh: An open, randomised controlled trial. The Lancet. 2015 Sep 12;386(9998):1057–65.

7. Milési C, Essouri S, Pouyau R, Liet JM, Afanetti M, Portefaix A, et al. High flow nasal cannula (HFNC) versus nasal continuous positive airway pressure (nCPAP) for the initial respiratory management of acute viral bronchiolitis in young infants: a multicenter randomized controlled trial (TRAMONTANE study). Intensive Care Med. 2017 Feb 1;43(2):209–16.

8. Peters MJ, Agbeko R, Davis P, Klein N, Zenasi Z, Jones A, et al. Randomised Study of early Continuous positive airways pressure in Acute Respiratory Failure in children with impaired immunity (SCARF). 2018;

9. Lalgudi Ganesan S, Jayashree M, Singhi SC, Bansal A. Airway pressure release ventilation in pediatric acute respiratory distress syndrome: A randomized controlled trial. Am J Respir Crit Care Med. 2018 Nov 1;198(9):1199–207.

10. Milési C, Pierre AF, Deho A, Pouyau R, Liet JM, Guillot C, et al. A multicenter randomized controlled trial of a 3-L/kg/min versus 2-L/kg/min high-flow nasal cannula flow rate in young infants with severe viral bronchiolitis (TRAMONTANE 2). Intensive Care Med. 2018 Nov 1;44(11):1870–8.

11. Montonati DM, Landry LM, Moreno GE, Vassallo JC, Galván E, Krynski M, et al. Estudio comparativo de dos modos de ventilacion no invasiva para retirar la asistencia respiratoria mecanica en lactantes post- operatorios de cirugia cardiovascular. Med Infant [Internet]. 2014;XXI:244–7. Available from: http://medicinainfantil.org.ar

12. Chorney SR, Patel RC, Boyd AE, Stow J, Schmitt MM, Lipman D, et al. Timing the First Pediatric Tracheostomy Tube Change: A Randomized Controlled Trial. Otolaryngology - Head and Neck Surgery (United States). 2021 Apr 1;164(4):869–76.

13. Borgi A, Louati A, Ghali N, Hajji A, Ayari A, Bouziri A, et al. High flow nasal cannula therapy versus continuous positive airway pressure and nasal positive pressure ventilation in infants with severe bronchiolitis: A randomized controlled trial. Pan African Medical Journal. 2021 Sep 1;40.

14. Kumar A, Joshi S, Tiwari N, Kumar V, Ramamurthy HR, Kumar G, et al. Comparative evaluation of high-flow nasal cannula oxygenation vs nasal intermittent ventilation in postoperative paediatric patients operated for acyanotic congenital cardiac defects. Med J Armed Forces India. 2022 Oct 1;78(4):454–62.

15. Bouve LR, Rozmus CL, Giordano P. Preparing Parents for Their Child’s Transfer from the PICU to the Pediatric Floor. Applied Nursing Research. 1999;12(3):114–20.

16. Melnyk BM, Alpert-Gillis LJ, Hensel PB, Cable-Beiling RC, Rubenstein JS. Helping Mothers Cope with a Critically Ill Child: A Pilot Test of the COPE Intervention. Res Nurs Health. 1997;20:3–14.

17. Melnyk BM, Alpert-Gillis L, Feinstein NF, Crean HF, Johnson J, Fairbanks E, et al. Creating opportunities for parent empowerment: program effects on the mental health/coping outcomes of critically ill young children and their mothers. Pediatrics. 2004;113(6).

18. Starks H, Doorenbos A, Lindhorst T, Bourget E, Aisenberg E, Oman N, et al. The Family Communication Study: A randomized trial of prospective pediatric palliative care consultation, study methodology and perceptions of participation burden. Contemp Clin Trials. 2016 Jul 1;49:15–20.

19. Michelson KN, Frader J, Charleston E, Rychlik K, Aniciete DY, Ciolino JD, et al. A Randomized Comparative Trial to Evaluate a PICU Navigator-Based Parent Support Intervention. Pediatric Critical Care Medicine. 2020 Sep 1;21(9):E617–27.

20. Murphy K, Mahmood N, Craven D, Gallagher J, Ross K, Speicher R, et al. Randomized pilot trial of ipratropium versus placebo in children with critical asthma. Pediatr Pulmonol. 2020 Dec 1;55(12):3287–92.

21. Krantz ID, Medne L, Weatherly JM, Wild KT, Biswas S, Devkota B, et al. Effect of Whole-Genome Sequencing on the Clinical Management of Acutely Ill Infants with Suspected Genetic Disease: A Randomized Clinical Trial. JAMA Pediatr. 2021;175(12):1218–26.

22. Willson DF, Thomas NJ, Markovitz BP, Bauman LA, Dicarlo J V, Pon S, et al. Effect of Exogenous Surfactant (Calfactant) in Pediatric Acute Lung Injury A Randomized Controlled Trial. JAMA [Internet]. 2005;293(4):470–6. Available from: http://jama.jamanetwork.com/

23. Agus MSD, Wypij D, Hirshberg EL, Srinivasan V, Faustino EV, Luckett PM, et al. Tight Glycemic Control in Critically Ill Children. New England Journal of Medicine. 2017 Feb 23;376(8):729–41.

24. Fink EL, Clark RSB, Berger RP, Fabio A, Angus DC, Watson RS, et al. 24 vs. 72 hours of hypothermia for pediatric cardiac arrest: A pilot, randomized controlled trial. Resuscitation. 2018 May 1;126:14–20.

25. Mondardini MC, Daverio M, Caramelli F, Conti G, Zaggia C, Lazzarini R, et al. Dexmedetomidine for prevention of opioid/benzodiazepine withdrawal syndrome in pediatric intensive care unit: Interim analysis of a randomized controlled trial. Pharmacotherapy. 2022 Feb 1;42(2):145–53.

26. Schlapbach LJ, Gibbons KS, Horton SB, Johnson K, Long DA, Buckley DHF, et al. Effect of Nitric Oxide via Cardiopulmonary Bypass on Ventilator-Free Days in Young Children Undergoing Congenital Heart Disease Surgery: The NITRIC Randomized Clinical Trial. Journal of American Medical Association. 2022 Jul 5;328(1):38–47.

27. Curley MAQ, Hibberd PL, Fineman LD, Wypij D, Shih MC, Thompson JE, et al. Effect of Prone Positioning on Clinical Outcomes in Children With Acute Lung Injury A Randomized Controlled Trial. J Am Med Assoc [Internet]. 2005;294(2):229–37. Available from: http://jama.jamanetwork.com/

28. Guerguerian AM, Gauthier M, Lebel MH, Farrell CA, Lacroix J. Ribavirin in Ventilated Respiratory Syncytial Virus Bronchiolitis A Randomized, Placebo-controlled Trial. Am J Respir Crit Care Med [Internet]. 1999;160:829–34. Available from: www.atsjournals.org

29. Katz SE, Crook J, Gillon J, Stanford JE, Wang L, Colby JM, et al. Use of a Procalcitonin-guided Antibiotic Treatment Algorithm in the Pediatric Intensive Care Unit. Pediatric Infectious Disease Journal. 2021 Apr 1;40(4):333–7.

30. Boyer L V, Theodorou AA, Berg RA, Mallie J, Chávez-Méndez A, García-Ubbelohde W, et al. Antivenom for Critically Ill Children with Neurotoxicity from Scorpion Stings A bs tr ac t. N Engl J Med. 2009;360(20):2090–8.

31. Simma B, Burger R, Falk M, Sacher P, Fanconi S. A prospective, randomized, and controlled study of fluid management in children with severe head injury: lactated Ringer’s solution versus hypertonic saline. Crit Care Med. 1998;26(7):1265–70.

32. Beca J, Mcsharry B, Erickson S, Yung M, Schibler A, Slater A, et al. Hypothermia for traumatic brain injury in children - A Phase II randomized controlled trial. Crit Care Med. 2015 Jul 21;43(7):1458–66.

33. Al-Biltagi MAM, Abo-Elezz AAE, Abd-Elhafez MA, Mabrouk MM, Suliman GA. Beneficial Effects of Omega-3 Supplement to the Enteral Feeding in Children with Mild to Moderate Sepsis. J Intensive Care Med. 2017 Mar 1;32(3):212–7.

34. Sarkar M, Sinha R, Roychowdhoury S, Mukhopadhyay S, Ghosh P, Dutta K. Comparative Study between Noninvasive Continuous Positive Airway Pressure and Hot Humidified High-flow Nasal Cannulae as a Mode of Respiratory Support in Infants with Acute Bronchiolitis in Pediatric Intensive Care Unit of a Tertiary Care Hospital. Indian Journal of Critical Care Medicine. 2018;22(2).

35. Esses SA. Post-Intensive Care Syndrome: Educational Interventions for Parents of Hospitalized Children. PICS Educational Interventions. 2017;

36. Adineh M, Toulabi T, Pournia Y, Baraz S. The Effect of Family Presence during Pediatric Intensive Care Unit Bedside on Family General Health: a Clinical Trial Study. Original Article [Internet]. 2016;4(29):1809–17. Available from: http://ijp.mums.ac.ir

37. Sahu MK, Bipin C, Niraghatam HV, Karanjkar A, Singh SP, Rajashekar P, et al. Vitamin D Deficiency and Its Response to Supplementation as “Stoss Therapy” in Children with Cyanotic Congenital Heart Disease Undergoing Open Heart Surgery. Journal of Cardiac Critical Care TSS. 2020 Aug;03(01):17–23.

38. Chandra S, Vijayshri, Gupta A, Goyal P, Prasad PL. High flow nasal cannula vs non-invasive ventilation in pediatric ARDS: an RCT. Pediatric Review: International Journal of Pediatric Research [Internet]. 2018; Available from: www.pediatricreview.in

39. Doymaz S, Ahmed YE, Francois D, Pinto R, Gist R, Steinberg M, et al. Methylprednisolone, dexamethasone or hydrocortisone for acute severe pediatric asthma: does it matter? Journal of Asthma. 2022;59(3):590–6.

40. Ramnarayan P, Richards-Belle A, Drikite L, Saull M, Orzechowska I, Darnell R, et al. Effect of High-Flow Nasal Cannula Therapy vs Continuous Positive Airway Pressure Following Extubation on Liberation From Respiratory Support in Critically Ill Children: A Randomized Clinical Trial. JAMA - Journal of the American Medical Association. 2022 Apr 26;327(16):1555–65.

41. Fernandes ICO, Fernandes JC, Cordeiro A, Hsin SH, Bousso A, Ejzenberg B, et al. Efficacy and safety of nebulized L-epinephrine associated with dexamethasone in postintubation laringitis. J Pediatr (Rio J). 2001;77(3):179–88.

42. Venter M, Rode H, Sive A, Visser M. Enteral resuscitation and early enteral feeding in children with major burns-Effect on McFarlane response to stress. Burns. 2007 Jun;33(4):464–71.

43. Gummalla P, Weaver D, Ahmed Y, Shah V, Keenaghan M, Doymaz S. Intravenous methylprednisolone versus intravenous methylprednisolone combined with inhaled budesonide in acute severe pediatric asthma. Journal of Asthma. 2021;58(11):1512–7.

44. Liet JM, Millotte B, Tucci M, Laflammme S, Hutchison J, Creery D, et al. Noninvasive therapy with helium-oxygen for severe bronchiolitis. Journal of Pediatrics. 2005 Dec;147(6):812–7.

45. Santana JC, Saldanha S, Barreto M, Piva JP, Garcia PC. Estudo controlado do uso endovenoso de sulfato de magnésio ou de salbutamol no tratamento precoce da crise de asma aguda grave em crianças Randomized clinical trial of intravenous magnesium sulfate versus salbutamol in the early management of severe acute asthma in children. J Pediatr (Rio J). 2001;77.

46. Esposito S, Garziano M, Rainone V, Trabattoni D, Biasin M, Senatore L, et al. Immunomodulatory activity of pidotimod administered with standard antibiotic therapy in children hospitalized for community-acquired pneumonia. J Transl Med. 2015 Sep 3;13(1).

47. Patil A, Bellad R. Effectiveness of 7% versus 3% hypertonic saline nebulization in infants with acute bronchiolitis : A randomized controlled trail. Journal of Pediatric Critical Care. 2019;6(5):21.

48. Klotz D, Seifert V, Baumgartner J, Teufel U, Fuchs H. High-flow nasal cannula vs standard respiratory care in pediatric procedural sedation: A randomized controlled pilot trial. Pediatr Pulmonol. 2020 Oct 1;55(10):2706–12.

49. Boeschoten SA, Buysse CMP, de Winter BCM, van Rosmalen J, de Jongste JC, de Jonge RC, et al. Efficacy of a loading dose of IV salbutamol in children with severe acute asthma admitted to a PICU: a randomized controlled trial. Eur J Pediatr. 2022 Oct 1;181(10):3701–9.

50. Buckingham SC, Jafri HS, Bush AJ, Carubelli CM, Sheeran P, Hardy RD, et al. A Randomized, Double-Blind, Placebo-Controlled Trial of Dexamethasone in Severe Respiratory Syncytial Virus (RSV) Infection: Effects on RSV Quantity and Clinical Outcome. J Infect Dis [Internet]. 2002;185:1222–30. Available from: https://academic.oup.com/jid/article/185/9/1222/936102

51. Zhao S. Nasal continuous positive airway pressure for the treatment of severe pneumonia in children from the plateau area: clinical analysis of 47 cases. Chinese Journal of Contemporary Pediatrics. 2010;12(3):226–7.

52. Xia YL, Yan WX, Chen H. Efficacy and safety of sildenafil in the treatment of high altitude heart disease associated with severe pulmonary arterial hypertension in children: A preliminary evaluation. Chinese Journal of Contemporary Pediatrics. 2014 Jul 15;16(7):745–8.

53. Malhotra D, Gurcoo S, Qazi S, Gupta S. Randomized Comparative Efficacy of Dexamethasone to Prevent Postextubation Upper Airway Complications in Children and Adults in ICU. Indian J Anaesth [Internet]. 2009;53(4):443–9. Available from: http://www.ijaweb.org

54. Behera SK, Zuccaro JC, Wetzel GT, Alejos JC. Nesiritide improves hemodynamics in children with dilated cardiomyopathy: A pilot study. Pediatr Cardiol. 2009 Jan;30(1):26–34.

55. Behrens R, Hofbeck M, Singer H, Scharf J, Rupprecht T. Frequency of stress lesions of the upper gastrointestinal tract in paediatric patients after cardiac surgery: effects of prophylaxis. Br Heart 1H. 1994;72:186–9.

56. Lopez-Herce J, Dorao P, Elola P, Delgado MA, Ruza F, Madero R, et al. Frequency and prophylaxis of upper gastrointestinal hemorrhage in critically ill children: a prospective study comparing the efficacy of almagate, ranitidine, and sucralfate. Crit Care Med. 1992;20(8):1082–9.

57. Natale JAE, Guerguerian AM, Joseph JG, McCarter R, Shao C, Slomine B, et al. Pilot study to determine the hemodynamic safety and feasibility of magnesium sulfate infusion in children with severe traumatic brain injury. Pediatric Critical Care Medicine. 2007 Jan;8(1):1–9.

58. Nguyen TC, Han YY, Kiss JE, Hall MW, Hassett AC, Jaffe R, et al. Intensive plasma exchange increases a disintegrin and metalloprotease with thrombospondin motifs-13 activity and reverses organ dysfunction in children with thrombocytopenia-associated multiple organ failure. Crit Care Med. 2008;36(10):2878–87.

59. Ridling DA, Martin LD, Bratton SL. Endotracheal suctioning with or without instillation of isotonic sodium chloride solution in critically ill children. American Journal of Critical Care. 2003 May 1;12(3):212–9.

60. Vardi A, Salem Y, Padeh S, Paret G, Barzilay Z. Is propofol safe for procedural sedation in children? A prospective evaluation of propofol versus ketamine in pediatric critical care*. Crit Care Med. 2002;30(6):1231–6.

61. Minoshima R, Kosugi S, Nishimura D, Ihara N, Seki H, Yamada T, et al. Intra- and postoperative low-dose ketamine for adolescent idiopathic scoliosis surgery: A randomized controlled trial. Acta Anaesthesiol Scand. 2015 Nov 1;59(10):1260–8.

62. Foncerrada G, Lima F, Clayton RP, Mlcak RP, Enkhbaatar P, Herndon DN, et al. Safety of nebulized epinephrine in smoke inhalation injury. Journal of Burn Care and Research. 2017;38(6):396–402.

63. Thomas NJ, Spear D, Wasserman E, Pon S, Markovitz B, Singh AR, et al. CALIPSO: A Randomized Controlled Trial of Calfactant for Acute Lung Injury in Pediatric Stem Cell and Oncology Patients. Biology of Blood and Marrow Transplantation. 2018 Dec 1;24(12):2479–86.

64. Van Driest SL, Wang L, McLemore MF, Bridges BC, Fleming GM, McGregor TL, et al. Acute kidney injury risk-based screening in pediatric inpatients: a pragmatic randomized trial. Pediatr Res. 2020 Jan 1;87(1):118–24.

65. Peters MJ, Woolfall K, Khan I, Deja E, Mouncey PR, Wulff J, et al. Permissive versus restrictive temperature thresholds in critically ill children with fever and infection: A multicentre randomized clinical pilot trial. Crit Care. 2019 Mar 7;23(1).

66. Peters MJ, Jones GAL, Wiley D, Wulff J, Ramnarayan P, Ray S, et al. Conservative versus liberal oxygenation targets in critically ill children: the randomised multiple-centre pilot Oxy-PICU trial. Intensive Care Med. 2018 Aug 1;44(8):1240–8.

67. Barbosa E, Moreira EA, Goes JE, Faintuch J. Pilot study with a glutamine-supplemented enteral formula in critically ill infants. Rev Hosp Clin Fac Med Sao Paulo. 1999;54(1):21–4.

68. Briassoulis G, Filippou O, Hatzi E, Papassotiriou I, Hatzis T. Early enteral administration of immunonutrition in critically ill children: Results of a blinded randomized controlled clinical trial. Nutrition. 2005 Jul;21(7–8):799–807.

69. Briassoulis G, Filippou O, Kanariou M, Papassotiriou I, Hatzis T. Temporal nutritional and inflammatory changes in children with severe head injury fed a regular or an immune-enhancing diet: A randomized, controlled trial. Pediatric Critical Care Medicine. 2006 Jan;7(1):56–62.

70. Kamat P, Favaloro-Sabatier J, Rogers K, Stockwell JA. Use of methylene blue spectrophotometry to detect subclinical aspiration in enterally fed intubated pediatric patients. Pediatric Critical Care Medicine. 2008;9(3):299–303.

71. Marin VB, Rodriguez-Osiac L, Schlessinger L, Villegas J, Lopez M, Castillo-Duran C. Controlled study of enteral arginine supplementation in burned children: impact on immunologic and metabolic status. Nutrition. 2006 Jul;22(7–8):705–12.

72. van Waardenburg DA, de Betue CT, Goudoever JB van, Zimmermann LJ, Joosten KF. Critically ill infants benefit from early administration of protein and energy-enriched formula: A randomized controlled trial. Clinical Nutrition. 2009 Jun;28(3):249–55.

73. Carcillo JA, Dean JM, Holubkov R, Berger J, Meert KL, Anand KJS, et al. The randomized comparative pediatric critical illness stress-induced immune suppression (CRISIS) prevention trial. Pediatric Critical Care Medicine. 2012 Mar;13(2):165–73.

74. Geukers VG, Li Z, Ackermans MT, Bos AP, Jinfeng L, Sauerwein HP. High-carbohydrate/low-protein-induced hyperinsulinemia does not improve protein balance in children after cardiac surgery. Nutrition. 2012 Jun;28(6):644–50.

75. Banupriya B, Biswal N, Srinivasaraghavan R, Narayanan P, Mandal J. Probiotic prophylaxis to prevent ventilator associated pneumonia (VAP) in children on mechanical ventilation: an open-label randomized controlled trial. Intensive Care Med. 2015 Apr 1;41(4):677–85.

76. Geukers VG, Dijsselhof ME, Jansen NJG, Breur JMPJ, Van Harskamp D, Schierbeek H, et al. The effect of short-term high versus normal protein intake on whole-body protein synthesis and balance in children following cardiac surgery: A randomized double-blind controlled clinical trial. Nutr J. 2015 Jul 28;14(1).

77. Fivez T, Kerklaan D, Mesotten D, Verbruggen S, Wouters PJ, Vanhorebeek I, et al. Early versus late parenteral nutrition in critically ill children. N Engl J Med. 2016 Aug 1;374(12):1111–22.

78. Yuan X, Qian SY, Li Z, Zhang ZZ. Effect of zinc supplementation on infants with severe pneumonia. World Journal of Pediatrics. 2016 May 1;12(2):166–9.

79. Prakash V, Parameswaran N, Biswal N. Early versus late enteral feeding in critically ill children: a randomized controlled trial. Vol. 42, Intensive Care Medicine. Springer Verlag; 2016. p. 481–2.

80. Zhang H, Gu Y, Mi YP, Jin Y, Fu W, Latour JM. High-energy nutrition in paediatric cardiac critical care patients: a randomized controlled trial. Nurs Crit Care. 2018 Mar 1;24(2):97–102.

81. Hauschild DB, Oliveira LDA, Farias MS, Barbosa E, Bresolin NL, Mehta NM, et al. Enteral Protein Supplementation in Critically Ill Children: A Randomized Controlled Pilot and Feasibility Study. Journal of Parenteral and Enteral Nutrition. 2019 Feb 1;43(2):281–9.

82. Zhang J, Cui YQ, Luo Y, Chen XX, Li J. Assessment of Energy and Protein Requirements in Relation to Nitrogen Kinetics, Nutrition, and Clinical Outcomes in Infants Receiving Early Enteral Nutrition Following Cardiopulmonary Bypass. Journal of Parenteral and Enteral Nutrition. 2020 Mar 1;45(3):553–66.

83. Yanni GN, Madjid AS, Hendarto A, Jusman SWA, Munasir Z, Satari HI, et al. A randomized controlled trial of high parenteral protein feeding in septic children: The role of tumor necrosis factor-alpha-308 polymorphism. Medical Journal of Indonesia. 2020 Mar 1;29(1):19–25.

84. Roudi F, Khademi G, Ranjbar G, Rafatpanah H, Esmaily H, Nematy M. Effects of high-dose selenium supplementation on oxidative stress and inflammatory markers in critically ill children after gastrointestinal surgery: A randomized clinical trial. Iran J Pediatr. 2020;30(4):1–10.

85. Brown AM, Irving SY, Pringle C, Allen C, Brown MF, Nett S, et al. Bolus gastric feeds improve nutrition delivery to mechanically ventilated pediatric medical patients: Results of the COntinuous vs BOlus multicenter trial. Journal of Parenteral and Enteral Nutrition. 2022 Jul 1;46(5):1011–21.

86. ÇAKIR SÇ, ÖZKAN T, ÖZGÜR T. A comparison of clinical, hepatic and immunological effects of three different parenteral lipid emulsions in children. The European Research Journal. 2020 Nov 4;6(6):600–8.

87. Chen X, Zhang M, Song Y, Luo Y, Wang L, Xu Z, et al. Early high-energy feeding in infants following cardiac surgery: A randomized controlled trial. Transl Pediatr. 2021 Oct 1;10(10):2439–48.

88. Huang XJ, Guo FF, Li F, Zhao JC, Fan YZ, Wang N, et al. Nutritional support in children with pneumonia on mechanical ventilation by short-peptide enteral nutrition formula. Chinese Journal of Contemporary Pediatrics. 2020 Nov 15;22(11):1209–14.

89. Saied A, El Borolossy RM, Ramzy MA, Sabri NA. Effect of zinc versus vitamin A supplementation on pediatric patients with community-acquired pneumonia. Front Pharmacol. 2022 Aug 30;13.

90. Yu XR, Xie WP, Liu JF, Wang LW, Cao H, Chen Q. Effect of the Addition of Human Milk Fortifier to Breast Milk on the Early Recovery of Infants After Congenital Cardiac Surgery. Front Pediatr. 2021 Apr 27;9.

91. Baldasso E, Garcia PCR, Piva JP, Branco RG, Tasker RC. Pilot safety study of low-dose vasopressin in non-septic critically ill children. Intensive Care Med. 2009 Feb;35(2):355–9.

92. Bettendorf M, Schmidt KG, Grulich-Henn J, Ulmer HE, Heinrich UE. Tri-iodothyronine treatment in children after cardiac surgery: a double-blind, randomised, placebo-controlled study. The Lancet. 2000;356:529–34.

93. Botrán M, López-Herce J, Mencía S, Urbano J, Solana MJ, García A. Enteral nutrition in the critically ill child: Comparison of standard and protein-enriched diets. Journal of Pediatrics. 2011;159(1):27–32.

94. Cesar RG, de Carvalho WB. L-Epinephrine and dexamethasone in postextubation airway obstruction: A prospective, randomized, double-blind placebo-controlled study. Int J Pediatr Otorhinolaryngol. 2009 Dec;73(12):1639–43.

95. Clancy RR, McGaurn SA, Goin JE, Hirtz DG, Norwood WI, Gaynor JW, et al. Allopurinol neurocardiac protection trial in infants undergoing heart surgery using deep hypothermic circulatory arrest. Pediatrics. 2001;108(1):61–70.

96. Hsin SH, Fontes AMS, Bousso A, Fernandes ICOF, Cordeiro AMG, Miyake RS, et al. Therapeutic of septic children with purpuric presentation with two antibiotic schedules. J Pediatr (Rio J). 1998;74(4):315–24.

97. Klein BS, Perloff WH, Maki DG. Reduction of nosocomial infection during pediatric intensive care by protective isolation. N Engl J Med. 1989;320(26):1714–21.

98. Laitinen P, Happonen JM, Sairanen H, Peltola K, Rautiainen P, Korpela R, et al. Amrinone Versus Dopamine-Nitroglycerin After Reconstructive Surgery for Complete Atrioventricular Septal Defect. J Cardiothorac Vasc Anesth. 1997;11(7):870–4.

99. Malley R, Devincenzo J, Ramilo O, Dennehy PH, Meissner HC, Gruber WC, et al. Reduction of Respiratory Syncytial Virus (RSV) in Tracheal Aspirates in Intubated Infants by Use of Humanized Monoclonal Antibody to RSV F Protein. J Infect Dis [Internet]. 1998;178:1555–61. Available from: https://academic.oup.com/jid/article/178/6/1555/844421

100. Mondal RK, Singhi SC, Chakrabarti A, Jayashree M. Randomized comparison between fluconazole and itraconazole for the treatment of candidemia in a pediatric intensive care unit: A preliminary study. Pediatric Critical Care Medicine. 2004;5(6):561–5.

101. Pedreira MLG, Kusahara DM, de Carvalho WB, Núez SC, Peterlini MAS. Oral care interventions and oropharyngeal colonization in children receiving mechanical ventilation. American Journal of Critical Care. 2009 Jul;18(4):319–28.

102. Schroth M, Plank C, Meißner U, Eberle KP, Weyand M, Cesnjevar R, et al. Hypertonic-hyperoncotic solutions improve cardiac function in children after open-heart surgery. Pediatrics. 2006 Jul;118(1).

103. Valoor HT, Singhi S, Jayashree M. Low-dose hydrocortisone in pediatric septic shock: An exploratory study in a third world setting. Pediatric Critical Care Medicine. 2009 Jan;10(1):121–5.

104. Jeschke MG, Barrow RE, Herndon DN. Recombinant human growth hormone treatment in pediatric burn patients and its role during the hepatic acute phase response. Pediatric Critical Care . 2000;

105. Jeschke MG, Kulp GA, Kraft R, Finnerty CC, Mlcak R, Lee JO, et al. Intensive insulin therapy in severely burned pediatric patients: A Prospective randomized trial. Am J Respir Crit Care Med. 2010 Aug 1;182(3):351–9.

106. Chopra A, Kumar V, Dutta A. Hypertonic versus normal saline as initial fluid bolus in pediatric septic shock. Indian J Pediatr. 2011 Jul;78(7):833–7.

107. Larsen BMK, Goonewardene LA, Joffe AR, Van Aerde JE, Field CJ, Olstad DL, et al. Pre-treatment with an intravenous lipid emulsion containing fish oil (eicosapentaenoic and docosahexaenoic acid) decreases inflammatory markers after open-heart surgery in infants: A randomized, controlled trial. Clinical Nutrition. 2012 Jun;31(3):322–9.

108. Ricci Z, Garisto C, Favia I, Vitale V, Di Chiara L, Cogo PE. Levosimendan infusion in newborns after corrective surgery for congenital heart disease: Randomized controlled trial. Intensive Care Med. 2012 Jul;38(7):1198–204.

109. De Carvalho Onofre PS, Da Luz Gonçalves Pedreira M, Peterlini MAS. Placement of peripherally inserted central catheters in children guided by ultrasound: A prospective randomized, and controlled trial. Pediatric Critical Care Medicine. 2012 Sep;13(5).

110. Kusahara DM, Peterlini MAS, Pedreira MLG. Oral care with 0.12% chlorhexidine for the prevention of ventilator-associated pneumonia in critically ill children: Randomised, controlled and double blind trial. Int J Nurs Stud. 2012 Nov;49(11):1354–63.

111. Marwali EM, Boom CE, Sakidjan I, Santoso A, Fakhri D, Kartini A, et al. Oral triiodothyronine normalizes triiodothyronine levels after surgery for pediatric congenital heart disease. Pediatric Critical Care Medicine. 2013;14(7):701–8.

112. Baranwal AK, Meena JP, Singhi SC, Muralidharan J. Dexamethasone pretreatment for 24 h versus 6 h for prevention of postextubation airway obstruction in children: A randomized double-blind trial. Intensive Care Med. 2014 Sep 1;40(9):1285–94.

113. Ricci Z, Haiberger R, Pezzella C, Garisto C, Favia I, Cogo P. Furosemide versus ethacrynic acid in pediatric patients undergoing cardiac surgery: A randomized controlled trial. Crit Care. 2015 Jan 7;19(1).

114. Nallasamy K, Jayashree M, Singhi S, Bansal A. Low-dose vs standard-dose insulin in pediatric diabetic ketoacidosis: A randomized clinical trial. JAMA Pediatr. 2014 Nov 1;168(11):999–1005.

115. Hammer GB, Lewandowski A, Drover DR, Rosen DA, Cohane C, Anand R, et al. Safety and Efficacy of Sodium Nitroprusside during Prolonged Infusion in Pediatric Patients. Pediatric Critical Care Medicine. 2015 Jun 21;16(5):397–403.

116. Ventura AMC, Shieh HH, Bousso A, Góes PF, Fernandes IDCFO, De Souza DC, et al. Double-blind prospective randomized controlled trial of dopamine versus epinephrine as first-line vasoactive drugs in pediatric septic shock. Crit Care Med. 2015;43(11):2292–302.

117. Baranwal AK, Murthy AS, Singhi SC. High-dose oral ambroxol for early treatment of pulmonary acute respiratory distress syndrome: An exploratory, randomized, controlled pilot trial. J Trop Pediatr. 2015 Oct 1;61(5):339–50.

118. Onan IS, Ozturk E, Yildiz O, Altin HF, Odemis E, Erek E. The effect of intravenous iloprost on pulmonary artery hypertension after paediatric congenital heart surgery. Interact Cardiovasc Thorac Surg. 2016 Feb 1;22(2):194–9.

119. Aziz F, Patil P. Role of Prophylactic Vitamin K in Preventing Antibiotic Induced Hypoprothrombinemia. Indian J Pediatr. 2015 Mar 12;82(4):363–7.

120. Dittrich MHM, Brunow De Carvalho W, Lopes Lavado E. Evaluation of the “early” Use of Albumin in Children with Extensive Burns: A Randomized Controlled Trial. Pediatric Critical Care Medicine. 2016 Jun 1;17(6):e280–6.

121. Friedberg MK, Schwartz SM, Zhang H, Chiu-Man C, Manlhiot C, Ilina M V., et al. Hemodynamic effects of sustained postoperative cardiac resynchronization therapy in infants after repair of congenital heart disease: Results of a randomized clinical trial. Heart Rhythm. 2017 Feb 1;14(2):240–7.

122. Sabri MR, Bigdelian H, Hosseinzadeh M, Ahmadi A, Ghaderian M, Shoja M. Comparison of the therapeutic effects and side effects of tadalafil and sildenafil after surgery in young infants with pulmonary arterial hypertension due to systemic-to-pulmonary shunts. Cardiol Young. 2017 Nov 1;27(9):1686–93.

123. Dhochak N, Jayashree M, Singhi S. A randomized controlled trial of one bag vs. two bag system of fluid delivery in children with diabetic ketoacidosis: Experience from a developing country. J Crit Care. 2018 Feb 1;43:340–5.

124. Sankar J, Ismail J, Sankar MJ, Suresh CPS, Meena RS. Fluid bolus over 15-20 versus 5-10 minutes each in the first hour of resuscitation in children with septic shock: A randomized controlled trial. Pediatric Critical Care Medicine. 2017 Oct 1;18(10):e435–45.

125. Marwali EM, Boom CE, Budiwardhana N, Fakhri D, Roebiono PS, Santoso A, et al. Oral Triiodothyronine for Infants and Children Undergoing Cardiopulmonary Bypass. Annals of Thoracic Surgery. 2017 Aug 1;104(2):688–95.

126. Talwar S, Bhoje A, Khadagawat R, Chaturvedi P, Sreenivas V, Makhija N, et al. Oral thyroxin supplementation in infants undergoing cardiac surgery: A double-blind placebo-controlled randomized clinical trial. Journal of Thoracic and Cardiovascular Surgery. 2018 Sep 1;156(3):1209-1217.e3.

127. Garisto C, Ricci Z, Tofani L, Benegni S, Pezzella C, Cogo P. Use of low-dose dexmedetomidine in combination with opioids and midazolam in pediatric cardiac surgical patients: Randomized controlled trial. Minerva Anestesiol. 2018 Sep 1;84(9):1053–62.

128. Mackie AS, Booth KL, Newburger JW, Gauvreau K, Huang SA, Laussen PC, et al. A randomized, double-blind, placebo-controlled pilot trial of triiodothyronine in neonatal heart surgery. Journal of Thoracic and Cardiovascular Surgery. 2005 Sep;130(3):810–6.

129. Pietroboni PF, Carvajal CM, Zuleta YI, Ortiz PL, Lucero YC, Drago M, et al. Landmark versus ultrasound-guided insertion of femoral venous catheters in the pediatric intensive care unit: An efficacy and safety comparison study. Med Intensiva. 2020 Mar 1;44(2):96–100.

130. Wang A, Cui C, Fan Y, Zi J, Zhang J, Wang G, et al. Prophylactic use of levosimendan in pediatric patients undergoing cardiac surgery: A prospective randomized controlled trial. Crit Care. 2019 Dec 30;23(1).

131. Batko I, Koscielniak-Merak B, Tomasik PJ, Kobylarz K. Lidocaine reduces sevoflurane consumption and improves recovery profile in children undergoing major spine surgery. Medical Science Monitor. 2020 Mar 21;26.

132. Chen J, Chen J, Yang J, Chen Y, Liang Y, Lin Y. Investigating the Efficacy of Hydrocolloid Dressing for Preventing Nasotracheal Tube-Related Pressure Injury in the PICU. Pediatric Critical Care Medicine. 2020 Sep 1;21(9):E752–8.

133. Anantasit N, Thasanthiah S, Lertbunrian R. Balanced salt solution versus normal saline solution as initial fluid re-suscitation in pediatric septic shock: A randomized, double-blind con-trolled trial. Crit Care Shock. 2020;23(4).

134. Sankar J, Lalitha A V., Rameshkumar R, Mahadevan S, Kabra SK, Lodha R. Use of Honey Versus Standard Care for Hospital-Acquired Pressure Injury in Critically Ill Children: A Multicenter Randomized Controlled Trial. Pediatric Critical Care Medicine. 2021 Jun 1;22(6):E349–62.

135. Peiravian F, Amirghofran AA, Ghamsari H, Emaminia A. Additive Effect of Phosphodiesterase Inhibitors in Control of Pulmonary Hypertension after Congenital Cardiac Surgery in Children. Iran Journal of Pediatrics [Internet]. 2013;23(1):19–26. Available from: http://ijp.tums.ac.ir

136. El-Ella SSA, El-Mekkawy MS, Selim AM. Stress ulcer prophylaxis for critically ill children: routine use needs to be re-examined. An Pediatr (Engl Ed) [Internet]. 2022;96:402–9. Available from: www.analesdepediatria.org

137. Chao T, Parry I, Palackic A, Sen S, Spratt H, Mlcak RP, et al. The effects of short bouts of ergometric exercise for severely burned children in intensive care: A randomized controlled trial. Clin Rehabil. 2022 Aug 1;36(8):1052–61.

138. Gulla KM, Sankar J, Jat KR, Kabra SK, Lodha R. Dexmedetomidine vs Midazolam for Sedation in Mechanically Ventilated Children: A Randomized Controlled Trial. Indian Pediatr. 2021;58:117–22.

139. Jothinath K, Balakrishnan S, Raju V, Menon S, Osborn J. Clinical efficacy of levosimendan vs milrinone in preventing low cardiac output syndrome following pediatric cardiac surgery. Ann Card Anaesth. 2021 Apr 1;24(2):217–23.

140. Kumar A, Tiwari N, Ramamurthy H, Kumar V, Kumar G. A prospective randomized clinical study of perioperative oral thyroid hormone treatment for children undergoing surgery for congenital heart diseases. Ann Pediatr Cardiol. 2021 Apr 1;14(2):170–7.

141. Lomivorotov V, Kornilov I, Boboshko V, Shmyrev V, Bondarenko I, Soynov I, et al. Effect of Intraoperative Dexamethasone on Major Complications and Mortality among Infants Undergoing Cardiac Surgery: The DECISION Randomized Clinical Trial. JAMA - Journal of the American Medical Association. 2020 Jun 23;323(24):2485–92.

142. Negi SL, Mandal B, Singh RS, Puri GD. Myocardial protection and clinical outcomes in Tetralogy of Fallot patients undergoing intracardiac repair: a randomized study of two cardioplegic techniques. Perfusion (United Kingdom). 2019 Sep 1;34(6):495–502.

143. Oh HW, Lee JH, Kim HC, Kim EH, Song IK, Kim HS, et al. The effect of 6% hydroxyethyl starch (130/0.4) on acute kidney injury in paediatric cardiac surgery: a prospective, randomised trial. Anaesthesia. 2018 Feb 1;73(2):205–15.

144. Prajapati M, Patel J, Patel H, Gandhi H, Singh G, Patel P. Assessment of the effect of two regimens of milrinone infusion in paediatric patients with pulmonary artery hypertension undergoing corrective cardiac procedure: A prospective observational study. Ann Pediatr Cardiol. 2022 Jul 1;15(4):358–63.

145. Rameshkumar R, Satheesh P, Jain P, Anbazhagan J, Abraham S, Subramani S, et al. Low-Dose (0.05 Unit/kg/hour) vs Standard-Dose (0.1 Unit/kg/hour) Insulin in the Management of Pediatric Diabetic Ketoacidosis: A Randomized Double-Blind Controlled Trial. Indian Pediatr. 2021;58:617–23.

146. Soliman R, Ragheb A. Assessment of the effect of two regimens of milrinone infusion in pediatric patients undergoing fontan procedure: A randomized study. Ann Card Anaesth. 2018 Apr 1;21(2):134–40.

147. Talwar S, Chatterjee S, Sreenivas V, Makhija N, Kapoor PM, Choudhary SK, et al. Comparison of del Nido and histidine-tryptophan-ketoglutarate cardioplegia solutions in pediatric patients undergoing open heart surgery: A prospective randomized clinical trial. Journal of Thoracic and Cardiovascular Surgery. 2019 Mar 1;157(3):1182-1192.e1.

148. Vignesh V, Rameshkumar R, Mahadevan S. Comparison of Phenytoin, Valproate and Levetiracetam in Pediatric Convulsive Status Epilepticus: A Randomized Double-blind Controlled Clinical Trial. Indian Pediatr. 2020;57:222–7.

149. Bigham MT, Jacobs BR, Monaco MA, Brilli RJ, Wells D, Conway EM, et al. Helium/oxygen-driven albuterol nebulization in the management of children with status asthmaticus: A randomized, placebo-controlled trial. Pediatric Critical Care Medicine. 2010;11(3):356–61.

150. Biswas AK, Bruce DA, Sklar FH, Bokovoy JL, Sommerauer JF. Treatment of acute traumatic brain injury in children with moderate hypothermia improves intracranial hypertension. Crit Care Med. 2002;30(12):2742–51.

151. Papo MC, Frank J, Thompson AE. A prospective, randomized study of continuous versus intermittent nebulized albuterol for severe status asthmaticus in children. Crit Care Med. 1993;21:1479–86.

152. Ream RS, Loftis LL, Albers GM, Becker BA, Lynch RE, Mink RB. Efficacy of IV Theophylline in Children With Severe Status Asthmaticus* clinical investigations in critical care 1480 Clinical Investigations. Chest. 2001;119:1480–8.

153. Wheeler DS, Jacobs BR, Kenreigh CA, Bean JA, Hutson TK, Brilli RJ. Theophylline versus terbutaline in treating critically ill children with status asthmaticus: A prospective, randomized, controlled trial. Pediatric Critical Care Medicine. 2005 Mar;6(2):142–7.

154. Basnet S, Mander G, Andoh J, Klaska H, Verhulst S, Koirala J. Safety, efficacy, and tolerability of early initiation of noninvasive positive pressure ventilation in pediatric patients admitted with status asthmaticus: A pilot study. Pediatric Critical Care Medicine. 2012 Jul;13(4):393–8.

155. Torres S, Sticco N, Bosch JJ, Iolster T, Siaba A, Rivarola MR, et al. Effectiveness of magnesium sulfate as initial treatment of acute severe asthma in children, conducted in a tertiary-level university hospital. A randomized, controlled trial. Arch Argent Pediatr. 2012 Jul;110(4):291–6.

156. Kudukis TM, Manthous CA, Schmidt GA, Hall JB, Wylam ME. Inhaled helium-oxygen revisited: Effect of inhaled helium-oxygen during the treatment of status asthmaticus in children. J Pediatr. 1994;130(2):217–24.

157. Turner A, Shann F, Delzoppo C, Henning R, Slater A, Beca J, et al. A multicentre, randomised, double-blind, placebo-controlled trial of aminophylline for bronchiolitis in infants admitted to intensive care. Critical Care and Resuscitation [Internet]. 2014;183(3):220–4. Available from: www.jficm.anzca.edu.au/aaccm/journal/publi-

158. Chidini G, Piastra M, Marchesi T, De Luca D, Napolitano L, Salvo I, et al. Continuous positive airway pressure with helmet versus mask in infants with bronchiolitis: An RCT. Pediatrics. 2015 Apr 1;135(4):e868–75.

159. Saied Y, Hamdy H, Bazaraa H, Rady H, Elanwary S. Magnesium sulphate as an adjuvant therapy in critically ill infants and children presenting with wheezy chest in addition to standard treatment. In: European Respiratory Journal. European Respiratory Society (ERS); 2018. p. PA2321.

160. Jacobs BR, Lyons K, Brilli RJ. Erythropoietin therapy in children with bronchiolitis and anemia*. Pediatric Critical Care Medicine [Internet]. 2003;4(1):44–8. Available from: www.pccmjournal.com

161. Luchetti M, Casiraghi G, Valsecchi R, Galassini E, Marraro G. Porcine-derived surfactant treatment of severe bronchiolitis. Acta Anaesthesiol Scand. 1998;42:805–10.

162. Luchetti M, Ferrero F, Gallini C, Natale A, Pigna A, Tortorolo L, et al. Multicenter, randomized, controlled study of porcine surfactant in severe respiratory syncytial virus-induced respiratory failure*. Pediatric Critical Care Medicine. 2002;3(3):261–8.

163. Meert KL, Sarnaik AP, Gelmini MJ, Lieh-Lai MW. Aerosolized ribavirin in mechanically ventilated children with respiratory syncytial virus lower respiratory tract disease: a prospective, double-blind, randomized trial. Crit Care Med. 1994;22(4):566–72.

164. Smith DW, Frankel LR, Mathers LH, Tang ATS, Ariagno RL, Prober CG. A controlled trial of aerosolized ribavirin in infants receiving mechanical ventilation for severe respiratory syncytial virus infection. N Engl J Med. 1991;325(1):24–9.

165. Tibby SM, Hatherill M, Wright SM, Wilson P, Postle AD, Murdoch IA. Exogenous Surfactant Supplementation in Infants with Respiratory Syncytial Virus Bronchiolitis. Am J Respir Crit Care Med [Internet]. 2000;162:1251–6. Available from: www.atsjournals.org

166. Van Woensel JBM, Vyas H. Dexamethasone in children mechanically ventilated for lower respiratory tract infection caused by respiratory syncytial virus: A randomized controlled trial. Crit Care Med. 2011;39(7):1779–83.

167. Van Woensel JBM, Van Aalderen WMC, De Weerd W, Jansen NJG, Van Gestel JPJ, Markhorst DG, et al. Dexamethasone for treatment of patients mechanically ventilated for lower respiratory tract infection caused by respiratory syncytial virus. Thorax. 2003 May 1;58(5):383–7.

168. Ergul AB, Calıskan E, Samsa H, Gokcek I, Kaya A, Zararsiz GE, et al. Using a high-flow nasal cannula provides superior results to OxyMask delivery in moderate to severe bronchiolitis: a randomized controlled study. Eur J Pediatr. 2018 Aug 1;177(8):1299–307.

169. Conesa-Segura E, Reyes-Dominguez SB, Ríos-Díaz J, Ruiz-Pacheco MÁ, Palazón-Carpe C, Sánchez-Solís M. Prolonged slow expiration technique improves recovery from acute bronchiolitis in infants: FIBARRIX randomized controlled trial. Clin Rehabil. 2019 Mar 1;33(3):504–15.

170. Kong M, Zhang WW, Sewell K, Gorman G, Kuo HC, Aban I, et al. Azithromycin Treatment vs Placebo in Children with Respiratory Syncytial Virus-Induced Respiratory Failure: A Phase 2 Randomized Clinical Trial. JAMA Netw Open. 2020 Apr 23;3(4).

171. Sachdev A, Vohra R, Gupta N, Gupta D, Gupta S. Comparison of high-flow nasal cannula and noninvasive positive pressure ventilation in children with acute bronchiolitis. Journal of Pediatric Critical Care. 2020;7(4):168.

172. Cesar RG, Bispo BRP, Felix PHCA, Modolo MCC, Souza AAF, Horigoshi NK, et al. High-Flow Nasal Cannula versus Continuous Positive Airway Pressure in Critical Bronchiolitis: A Randomized Controlled Pilot. J Pediatr Intensive Care. 2020 Dec;09(04):248–55.

173. Gelbart B, McSharry B, Delzoppo C, Erickson S, Lee K, Butt W, et al. Pragmatic Randomized Trial of Corticosteroids and Inhaled Epinephrine for Bronchiolitis in Children in Intensive Care. Journal of Pediatrics. 2022 May 1;244:17-23.e1.

174. Berens RJ, Meyer MT, Mikhailov TA, Colpaert KD, Czarnecki ML, Ghanayem NS, et al. A prospective evaluation of opioid weaning in opioid-dependent pediatric critical care patients. Anesth Analg. 2006;102(4):1045–50.

175. Bogie AL, Towne D, Luckett PM, Abramo TJ, Wiebe RA. Comparison of Intravenous Terbutaline Versus Normal Saline in Pediatric Patients on Continuous High-Dose Nebulized Albuterol for Status Asthmaticus. Pediatr Emerg Care. 2007;23(6):355–61.

176. Darnell CM, Thompson J, Stromberg D, Roy L, Sheeran P. Effect of low-dose naloxone infusion on fentanyl requirements in critically ill children. Pediatrics. 2008 May;121(5):e1363–71.

177. Randolph AG, Wypij D, Venkataraman ST, Hanson JH, Gedeit RG, Meert KL, et al. Effect of Mechanical Ventilator Weaning Protocols on Respiratory Outcomes in Infants and Children A Randomized Controlled Trial. Journal of American Medical Association [Internet]. 2002;288:2561–8. Available from: https://jamanetwork.com/

178. Schroeder AR, Axelrod DM, Silverman NH, Rubesova E, Merkel E, Roth SJ. A continuous heparin infusion does not prevent catheter-related thrombosis in infants after cardiac surgery. Pediatric Critical Care Medicine. 2010;11(4):489–95.

179. Sorce LR, Hamilton SM, Gauvreau K, Mets MB, Hunter DG, Rahmani B, et al. Preventing corneal abrasions in critically ill children receiving neuromuscular blockade: A randomized, controlled trial. Pediatric Critical Care Medicine. 2009 Mar 1;10(2).

180. Willson DF, Md ;, Zaritsky A, Bauman LA, Dockery KMJ, Ms RL, et al. Instillation of calf lung surfactant extract (calfactant) is beneficial in pediatric acute hypoxemic respiratory failure Author Information. Critical Care Medicine Issue [Internet]. 1999;27(1):188–95. Available from: http://ovidsp.tx.ovid.com.proxy.bib.uottawa.ca/sp-3.17.0a/ovidweb.cgi

181. Bowens CD, Thompson JA, Thompson MT, Breitzka RL, Thompson DG, Sheeran PW. A trial of methadone tapering schedules in pediatric intensive care unit patients exposed to prolonged sedative infusions. Pediatric Critical Care Medicine. 2011;12(5):504–11.

182. Kline AM, Sorce L, Sullivan C, Weishaar J, Steinhorn DM. Use of a noninvasive electromagnetic device to place transpyloric feeding tubes in critically ill children. American Journal of Critical Care. 2011 Nov 1;20(6):453–60.

183. Adelson PD, Ragheb J, Muizelaar JP, Kanev P, Brockmeyer D, Beers SR, et al. Phase II clinical trial of moderate hypothermia after severe traumatic brain injury in children. Neurosurgery. 2005 Apr;56(4):740–53.

184. Hoffman TM, Wemovsky G, Atz AM, Kulik TJ, Nelson DP, Chang AC, et al. Efficacy and safety of milrinone in preventing low cardiac output syndrome in infants and children after corrective surgery for congenital heart disease. Circulation. 2003 Feb 25;107(7):996–1002.

185. Thomas NJ, Guardia CG, Moya FR, Cheifetz IM, Markovitz B, Cruces P, et al. A pilot, randomized, controlled clinical trial of lucinactant, a peptide-containing synthetic surfactant, in infants with acute hypoxemic respiratory failure. Pediatric Critical Care Medicine. 2012 Nov;13(6):646–53.

186. Jacobs BR, Nadkarni V, Goldstein B, Checchia P, Ayad O, Bean J, et al. Nutritional immunomodulation in critically ill children with acute lung injury: Feasibility and impact on circulating biomarkers. Pediatric Critical Care Medicine. 2013 Jan;14(1).

187. Milstone AM, Elward A, Song X, Zerr DM, Orscheln R, Speck K, et al. Daily chlorhexidine bathing to reduce bacteraemia in critically ill children: A multicentre, cluster-randomised, crossover trial. The Lancet. 2013 Mar 1;381(9872):1099–106.

188. Levin M, Quint PA, Goldstein B, Barton P, Bradley JS, Shemie SD, et al. Recombinant bactericidal/permeability-increasing protein (rBPI21) as adjunctive treatment for children with severe meningococcal sepsis: a randomised trial. rBPI21 Meningococcal Sepsis Study Group.

189. Madani S, Kauffman R, Simpson P, Lehr VT, Lai ML, Sarniak A, et al. Pharmacokinetics and pharmacodynamics of famotidine and ranitidine in critically ill children. J Clin Pharmacol. 2014;54(2):201–5.

190. Adelson PD, Wisniewski SR, Beca J, Brown SD, Bell M, Muizelaar JP, et al. Comparison of hypothermia and normothermia after severe traumatic brain injury in children (Cool Kids): A phase 3, randomised controlled trial. Lancet Neurol. 2013 Jun;12(6):546–53.

191. Costello JM, Dunbar-Masterson C, Allan CK, Gauvreau K, Newburger JW, McGowan FX, et al. Impact of empiric nesiritide or milrinone infusion on early postoperative recovery after fontan surgery a randomized, double-blind, placebo-controlled trial. Circ Heart Fail. 2014 Jul 1;7(4):596–604.

192. Bronicki RA, Fortenberry J, Schreiber M, Checchia PA, Anas NG. Multicenter randomized controlled trial of inhaled nitric oxide for pediatric acute respiratory distress syndrome. Journal of Pediatrics. 2015 Feb 1;166(2):365-369.e1.

193. Curley MAQ, Wypij D, Watson RS, Grant MJC, Asaro LA, Cheifetz IM, et al. Protocolized sedation vs usual care in pediatric patients mechanically ventilated for acute respiratory failure: A randomized clinical trial. JAMA - Journal of the American Medical Association. 2015 Jan 27;313(4):379–89.

194. Drago BB, Kimura D, Rovnaghi CR, Schwingshackl A, Rayburn M, Meduri GU, et al. Double-Blind, Placebo-Controlled Pilot Randomized Trial of Methylprednisolone Infusion in Pediatric Acute Respiratory Distress Syndrome. Pediatric Critical Care Medicine. 2015 Mar 20;16(3):e74–81.

195. Axelrod DM, Sutherland SM, Anglemyer A, Grimm PC, Roth SJ. A Double-Blinded, Randomized, Placebo-Controlled Clinical Trial of Aminophylline to Prevent Acute Kidney Injury in Children Following Congenital Heart Surgery with Cardiopulmonary Bypass. Pediatric Critical Care Medicine. 2016 Feb 1;17(2):135–43.

196. Shein SL, Gallagher JT, Deakins KM, Weinert DM. Prophylactic use of nebulized hypertonic saline in mechanically ventilated children: A randomized blinded pilot study. Respir Care. 2016 May 1;61(5):586–92.

197. Gottschlich MM, Mayes T, Khoury J, Kagan RJ. Clinical trial of Vitamin D2 vs D3 supplementation in critically ill pediatric burn patients. Journal of Parenteral and Enteral Nutrition. 2017 Mar 1;41(3):412–21.

198. Kwiatkowski DM, Goldstein SL, Cooper DS, Nelson DP, Morales DLS, Krawczeski CD. Peritoneal Dialysis vs Furosemide for prevention of fluid overload in infants after cardiac surgery a randomized clinical trial. JAMA Pediatr. 2017 Apr 1;171(4):357–64.

199. Clark RSB, Empey PE, Bayir H, Rosario BL, Poloyac SM, Kochanek PM, et al. Phase I randomized clinical trial of N-acetylcysteine in combination with an adjuvant probenecid for treatment of severe traumatic brain injury in children. PLoS One. 2017 Jul 1;12(7).

200. Brown AM, Fisher E, Forbes ML. Bolus vs Continuous Nasogastric Feeds in Mechanically Ventilated Pediatric Patients: A Pilot Study. Journal of Parenteral and Enteral Nutrition. 2019 Aug 1;43(6):750–8.

201. Hill KD, Maharaj AR, Li JS, Thompson E, Barker PCA, Hornik CP. A Randomized, Controlled Pharmacokinetic and Pharmacodynamics Trial of Ambrisentan After Fontan Surgery. Pediatric Critical Care Medicine. 2020 Sep 1;21(9):E795–803.

202. Jha P, Rupp L, Bonilla L, Gelfond J, Shah JN, Meyer AD. Electromagnetic Versus Blind Guidance of a Postpyloric Feeding Tube in Critically Ill Children. Pediatrics [Internet]. 2020;146(4). Available from: http://publications.aap.org/pediatrics/article-pdf/146/4/e20193773/1080169/peds_20193773.pdf?casa_token=dv26JKl_Xz0AAAAA:PwqjlEnkXs7F8eWHHZ5C8y-7q7HJsBq0F-clCZjCPIpSoR0iQebgNaA_I0eUk10sVvGXMGjfPB4

203. Faustino EVS, Shabanova V, Raffini LJ, Kandil SB, Li S, Pinto MG, et al. Efficacy of Early Prophylaxis Against Catheter-Associated Thrombosis in Critically Ill Children: A Bayesian Phase 2b Randomized Clinical Trial. Crit Care Med. 2021 Mar 1;49(3):E235–46.

204. Hymel KP, Armijo-Garcia V, Musick M, Marinello M, Herman BE, Weeks K, et al. A Cluster Randomized Trial to Reduce Missed Abusive Head Trauma in Pediatric Intensive Care Settings. In: Journal of Pediatrics. Mosby Inc.; 2021. p. 260-268.e3.

205. Nadel S, Goldstein B, Williams MD, Dalton H, Peters M, Macias WL, et al. Articles Drotrecogin alfa (activated) in children with severe sepsis: a multicentre phase III randomised controlled trial. www.thelancet.com [Internet]. 2007;369:836–43. Available from: www.thelancet.com

206. Perondi MBM, Reis AG, Paiva EF, Nadkarni VM, Berg RA. A Comparison of High-Dose and Standard-Dose Epinephrine in Children with Cardiac Arrest. N Engl J Med [Internet]. 2004;17(22):1722–52. Available from: www.nejm.org

207. Rodríguez-Moya VS, Gallo-Borrero CM, Santos-Áreas D, Prince-Martínez IA, Díaz-Casañas E, López-Herce Cid J. Exogenous surfactant and alveolar recruitment in the treatment of the acute respiratory distress syndrome. Clinical Respiratory Journal. 2017 Nov 1;11(6):1032–9.

208. Ullman AJ, Long D, Williams T, Pearson K, Mihala G, Mattke AC, et al. Innovation in Central Venous Access Device Security: A Pilot Randomized Controlled Trial in Pediatric Critical Care. Pediatric Critical Care Medicine. 2019 Oct 1;20(10):E480–8.

209. McNally JD, O’Hearn K, Fergusson DA, Lougheed J, Doherty DR, Maharajh G, et al. Prevention of post-cardiac surgery vitamin D deficiency in children with congenital heart disease: a pilot feasibility dose evaluation randomized controlled trial. Pilot Feasibility Stud. 2020 Dec 1;6(1).

210. Meert KL, Daphtary KM, Metheny NA. Gastric vs Small-Bowel Feeding in Critically Ill Children Receiving Mechanical Ventilation* A Randomized Controlled Trial. Chest [Internet]. 2004;126(3):872–8. Available from: www.chestjournal.org

211. Spalding HK, Sullivan KJ, Soremi O, Gonzalez F, Goodwin SR. Bedside placement of transpyloric feeding tubes in the pediatric intensive care unit using gastric insufflation. Crit Care Med. 2000;28(6):2041–4.

212. Sahu MK, Singal A, Menon R, Singh SP, Mohan A, Manral M, et al. Early enteral nutrition therapy in congenital cardiac repair postoperatively: A randomized, controlled pilot study. Ann Card Anaesth. 2016 Oct 1;19(4):653–61.

213. Cui Y, Li L, Hu C, Shi H, Li J, Gupta RK, et al. Effects and Tolerance of Protein and Energy-Enriched Formula in Infants Following Congenital Heart Surgery: A Randomized Controlled Trial. Journal of Parenteral and Enteral Nutrition. 2018 Jan 1;42(1):196–204.

214. Floh AA, Herridge J, Fan CPS, Manlhiot C, Mccrindle BW, Van Arsdell G, et al. Rapid Advancement in Enteral Nutrition Does Not Affect Systemic Inflammation and Insulin Homeostasis Following Pediatric Cardiopulmonary Bypass Surgery. Pediatric Critical Care Medicine. 2020 Jul 1;21(7):E441–8.

215. Tan Q, Wang Y, Zhang G, Lu B, Wang T, Tao T, et al. The metabolic effects of multi-trace elements on parenteral nutrition for critically ill pediatric patients: a randomized controlled trial and metabolomic resesarch. Transl Pediatr. 2021;10(10):2579–93.

216. Carman B, Cahill T, Warden G, Mccall J. A Prospective, Randomized Comparison of the Volume Diffusive Respirator® vs Conventional Ventilation for Ventilation of Burned Children 2001 ABA Paper. Journal of Burn Care & Rehabilitation [Internet]. 2002;23(6):444–8. Available from: https://academic.oup.com/jbcr/article/23/6/444/4733801

217. Rodríguez JA, Von Dessauer B, Duffau G. Non-invasive continuous positive airways pressure for laryngitis postextubation in pediatric patients. Arch Bronconeumol. 2002;38(10):463–7.

218. Kallio M, Peltoniemi O, Anttila E, Pokka T, Kontiokari T. Neurally adjusted ventilatory assist (NAVA) in pediatric intensive care - A randomized controlled trial. Pediatr Pulmonol. 2015 Jan 1;50(1):55–62.

219. El-Nawawy A, Moustafa A, Heshmat H, Abouahmed A. High frequency oscillatory ventilation versus conventional mechanical ventilation in pediatric acute respiratory distress syndrome: A randomized controlled study. Turkish Journal of Pediatrics. 2017;59(2):130–43.

220. Ferreira F V., Sugo EK, Aragon DC, Carmona F, Carlotti APCP. Spontaneous Breathing Trial for Prediction of Extubation Success in Pediatric Patients Following Congenital Heart Surgery: A Randomized Controlled Trial. Pediatric Critical Care Medicine. 2019 Oct 1;20(10):940–6.

221. Enayati F, Amini S, Gerdrodbari MG, Jarahi L, Ansari M. Effect of high-flow nasal Oxygen on respiratory parameters and pulmonary complications after early extubation following pediatric heart surgery. Journal of Comprehensive Pediatrics. 2021 Aug 1;12(3).

222. Jeschke MG, Finnerty CC, Kulp GA, Przkora R, Mlcak RP, Herndon DN. Combination of recombinant human growth hormone and propranolol decreases hypermetabolism and inflammation in severely burned children. Pediatric Critical Care Medicine. 2008;9(2):209–16.

223. Mecott GA, Herndon DN, Kulp GA, Brooks NC, Al-Mousawi AM, Kraft R, et al. The use of exenatide in severely burned pediatric patients. Crit Care. 2010 Aug 11;14(4).

224. Rey C, Los-Arcos M, Hernández A, Sánchez A, Díaz JJ, López-Herce J. Hypotonic versus isotonic maintenance fluids in critically ill children: A multicenter prospective randomized study. Acta Paediatrica, International Journal of Paediatrics. 2011 Aug;100(8):1138–43.

225. Rouine-Rapp K, Mello DM, Hanley FL, Reddy VM, Soifer S. Effect of enalaprilat on postoperative hypertension after surgical repair of coarctation of the aorta. Pediatric Critical Care Medicine. 2003;4(3):327–32.

226. Brutocao D, Bratton SL, Thomas JR, Schrader PF, Coles PG, Lynn AM. Comparison of Hetastarch With Albumin for Postoperative Volume Expansion in Children After Cardiopulmonary Bypass. J Cardiothorac Vasc Anesth. 1996;10(3):348–51.

227. Aydogan MS, Korkmaz MF, Ozgül U, Erdogan MA, Yucel A, Karaman A, et al. Pain, fentanyl consumption, and delirium in adolescents after scoliosis surgery: Dexmedetomidine vs midazolam. Paediatr Anaesth. 2013 May;23(5):446–52.

228. Sun CY, Lee KC, Lin IH, Wu CL, Huang HP, Lin YY, et al. Near-infrared light device can improve intravenous cannulation in critically ill children. Pediatr Neonatol. 2013 Jun;54(3):194–7.

229. Ebade AA, Khalil MA, Mohamed AK. Levosimendan is superior to dobutamine as an inodilator in the treatment of pulmonary hypertension for children undergoing cardiac surgery. J Anesth. 2013 Jun;27(3):334–9.

230. Kumar R, Singhi S, Singhi P, Jayashree M, Bansal A, Bhatti A. Randomized controlled trial comparing cerebral perfusion pressure-targeted therapy versus intracranial pressure-targeted therapy for raised intracranial pressure due to acute CNS infections in children. Crit Care Med. 2014;42(8):1775–87.

231. Abd-Elshafy SK, Khalaf GS, Abo-Kerisha MZ, Ahmed NT, El-Aziz MAA, Mohamed MA. Not All Sounds Have Negative Effects on Children Undergoing Cardiac Surgery. J Cardiothorac Vasc Anesth. 2015 Oct 1;29(5):1277–84.

232. Prasertsan P, Nakju D, Lertbunrian R, Chantra M, Anantasit N. Nebulized fluticasone for preventing postextubation stridor in intubated children: A randomized, double-blind placebo-controlled trial∗. Pediatric Critical Care Medicine. 2017 May 1;18(5):e201–6.

233. Bhasin S, Gogia P, Nair R, Sahoo TK. Perioperative sildenafil therapy for children with ventricular septal defects and associated pulmonary hypertension undergoing corrective surgery: A randomised clinical trial. Indian J Anaesth. 2017 Oct 1;61(10):798–802.

234. EL-Nawawy AA, Abdelmohsen AM, Hassouna HM. Role of echocardiography in reducing shock reversal time in pediatric septic shock: a randomized controlled trial. J Pediatr (Rio J). 2018 Jan 1;94(1):31–9.

235. Song IK, Kim EH, Lee JH, Kang P, Kim HS, Kim JT. Utility of Perioperative Lung Ultrasound in Pediatric Cardiac Surgery: A Randomized Controlled Trial. Anesthesiology. 2018 Apr 1;128(4):718–27.

236. Razavi Z, Maher S, Fredmal J. Comparison of subcutaneous insulin aspart and intravenous regular insulin for the treatment of mild and moderate diabetic ketoacidosis in pediatric patients. Endocrine. 2018 Aug 1;61(2):267–74.

237. Lee B, Park JD, Choi YH, Han YJ, Suh DI. Efficacy and safety of fentanyl in combination with midazolam in children on mechanical ventilation. J Korean Med Sci. 2019 Jan 1;34(3).

238. Takeshita J, Yoshida T, Nakajima Y, Nakayama Y, Nishiyama K, Ito Y, et al. Dynamic Needle Tip Positioning for Ultrasound-Guided Arterial Catheterization in Infants and Small Children With Deep Arteries: A Randomized Controlled Trial. J Cardiothorac Vasc Anesth. 2019 Jul 1;33(7):1919–25.

239. Takeshita J, Yoshida T, Nakajima Y, Nakayama Y, Nishiyama K, Ito Y, et al. Superiority of Dynamic Needle Tip Positioning for Ultrasound-Guided Peripheral Venous Catheterization in Patients Younger Than 2 Years Old: A Randomized Controlled Trial. Pediatric Critical Care Medicine. 2019 Sep 1;20(9):E410–4.

240. Takeshita J, Nakajima Y, Kawamura A, Taniguchi M, Shimizu Y, Takeuchi M, et al. Ultrasonographic Detection of Micro-Bubbles in the Right Atrium to Confirm Peripheral Venous Catheter Position in Children. Crit Care Med. 2019 Oct 1;47(10):E836–40.

241. Rameshkumar R, Bansal A, Singhi S, Singhi P, Jayashree M. Randomized Clinical Trial of 20% Mannitol Versus 3% Hypertonic Saline in Children With Raised Intracranial Pressure Due to Acute CNS Infections. Pediatric Critical Care Medicine. 2020;21:1071–80.

242. Macaire P, Ho N, Nguyen V, Phan Van H, Dinh Nguyen Thien K, Bringuier S, et al. Bilateral ultrasound-guided thoracic erector spinae plane blocks using a programmed intermittent bolus improve opioid-sparing postoperative analgesia in pediatric patients after open cardiac surgery: A randomized, double-blind, placebo-controlled trial. Reg Anesth Pain Med. 2020 Oct 1;45(10):805–12.

243. Abbaskhanian A, Sheidaee K, Charati JY. Comparison of the effect of continuous intravenous infusion of sodium valproate and midazolam on management of status epilepticus in children. Archives de Pediatrie. 2021 Nov 1;28(8):696–701.

244. Attia WA, Mohamed O, Aziz A, Mohamed A, Reheem A, Mohamed Ezz-Eldin Sayed O, et al. Comparison Between Sedative And Analgesic Effects Of Dexmedetomidine Versus Fentanyl For Pediatric Patients Following Cardiac Surgery In Intensive Care Unit. J Pharm Negat Results. 2022;13:2022.

245. Downey LA, Andrews J, Hedlin H, Kamra K, McKenzie ED, Hanley FL, et al. Fibrinogen Concentrate as an Alternative to Cryoprecipitate in a Postcardiopulmonary Transfusion Algorithm in Infants Undergoing Cardiac Surgery: A Prospective Randomized Controlled Trial. Anesth Analg. 2020 Mar 1;130(3):740–51.

246. Karakaya Z, Duyu M, Yersel MN. Oral mucosal mouthwash with chlorhexidine does not reduce the incidence of ventilator-associated pneumonia in critically ill children: A randomised controlled trial. Australian Critical Care. 2022 Jul 1;35(4):336–44.

247. Karri S, Mandal B, Kumar B, Puri G, Thingnam S, Kumar H, et al. Effect of perioperative use of oral triidothyronine for infants undergoing complex congenital cardiac surgeries under cardiopulmonary bypass: A double-blinded randomised controlled study. Ann Card Anaesth. 2022 Jul 1;25(3):270–8.

248. Keskin H, Keskin F, Aydin P, Guler MA, Ahiskalioglu A. Syringe-Free, Long-Axis in-Plane Versus Short-Axis Classic out-of-Plane Approach for Ultrasound-Guided Internal Jugular Vein Catheter Placement in Critically Ill Children: A Prospective Randomized Study. J Cardiothorac Vasc Anesth. 2021 Jul 1;35(7):2094–9.

249. Kim EH, Lee JH, Kim HS, Jang YE, Ji SH, Kim WH, et al. Effects of intraoperative dexmedetomidine on the incidence of acute kidney injury in pediatric cardiac surgery patients: A randomized controlled trial. Paediatr Anaesth. 2020 Oct 1;30(10):1132–8.

250. Sheriff A, Rameshkumar R, Chidambaram M, Maulik K, Kumar RS, Jamal A, et al. Epinephrine Plus Vasopressin vs Epinephrine Plus Placebo in Pediatric Intensive Care Unit Cardiopulmonary Resuscitation: A Randomized Double Blind Controlled Clinical Trial. Indian Pediatr. 2021;58:624–30.

251. Siddiqui H, Siddiqui SA, Yadav RK, Singh MV, Kumar D, Kumar D, et al. Nebulized Salbutamol with or without Magnesium Sulphate in the Management of Acute Asthma in Children in India: A Randomized Controlled Trial. J Trop Pediatr. 2022 Oct 1;68(5).

252. Xu Y, Li L, Hou J, Zhang N, Zeng M, Qiu Q, et al. 3D CT airway evaluation-guided intraluminal placement of endobronchial blocker in pediatric patients: a randomized controlled study. Transl Pediatr. 2021 Mar 1;10(3):625–34.

253. Zhang JQ, Yang QY, Xue FS, Zhang W, Yang GZ, Liao X, et al. Preoperative oral thyroid hormones to prevent euthyroid sick syndrome and attenuate myocardial ischemia-reperfusion injury after cardiac surgery with cardiopulmonary bypass in children: A randomized, double-blind, placebo-controlled trial. Medicine (United States). 2018 Sep 1;97(36).

254. Zhang X, Chang L, Pan SD, Yan FX. Dexmedetomidine Improves Non-rapid Eye Movement Stage 2 Sleep in Children in the Intensive Care Unit on the First Night After Laparoscopic Surgery. Front Pediatr. 2022 Apr 27;10.

255. Cam B V., Tuan DT, Fonsmark L, Poulsen A, Tien NM, Tuan HM, et al. Randomized Comparison of Oxygen Mask Treatment vs. Nasal Continuous Positive Airway Pressure in Dengue Shock Syndrome with Acute Respiratory Failure. J Trop Pediatr. 2002;48:335–9.

256. Cifra HL, Velasco JNJ. A comparative study of the efficacy of 6% Haes-Steril and Ringer’s lactate in the management of dengue shock syndrome. Crit Care Shock. 2003;6(2):95–100.

257. Mehta V, Singhi P, Singhi S. Intravenous sodium valproate versus diazepam infusion for the control of refractory status epilepticus in children: A randomized controlled trial. J Child Neurol. 2007 Oct;22(10):1191–7.

258. Upadhyay M, Singhi S, Murlidharan J, Kaur N, Majumdar S. Randomized Evaluation of Fluid Resuscitation with Crystalloid (saline) and Colloid (polymer from degraded Gelatin in saline) in Pediatric Septic Shock †. Indian Pediatr. 2005;42:223–31.

259. Dung NM, Day NPJ, Tam DTH, Loan HT, Chau HTT, Minh LN, et al. Fluid Replacement in Dengue Shock Syndrome: A Randomized, Double-Blind Comparison of Four Intravenous-Fluid Regimens. Clinical Infectious Diseases. 1999;29:787–94.

260. Sebastian MR, Lodha R, Kapil A, Kabra SK. Oral mucosal decontamination with chlorhexidine for the prevention of ventilator-associated pneumonia in children-A randomized, controlled trial. Pediatric Critical Care Medicine. 2012 Sep;13(5).

261. Solana MJ, López-Herce J, Sánchez A, Sánchez C, Urbano J, López D, et al. 0.5 mg/kg versus 1 mg/kg of intravenous omeprazole for the prophylaxis of gastrointestinal bleeding in critically Ill children: A randomized study. Journal of Pediatrics. 2013;162(4).

262. Raksha SK, Dakshayani B, Premalatha R. Full volume isotonic (0.9%) vs. Two-thirds volume hypotonic (0.18%) intravenous maintenance fluids in preventing hyponatremia in children admitted to pediatric intensive care unit-A randomized controlled study. J Trop Pediatr. 2017 Dec 1;63(6):454–60.

263. Sankar J, Singh M, Kumar K, Sankar MJ, Kabra SK, Lodha R. ‘Intermittent’ versus ‘continuous’ ScvO2 monitoring in children with septic shock: a randomised, non-inferiority trial. Intensive Care Med. 2020 Jan 1;46(1):82–92.

264. Sachdev A, Sagar N, Gupta D, Gupta N, Gupta S. Efficacy of adjuvant colistimethate sodium nebulization in ventilator associated pneumonia in pediatric intensive care unit - A randomized controlled study. Journal of Pediatric Critical Care. 2019;6(3):15.

265. Souza GSB, Novais MFM, Lemes GE, de Mello MLFMF, de Sales SCD, da Costa Cunha K, et al. Effectiveness of Different Physiotherapy Protocols in Children in the Intensive Care Unit: A Randomized Clinical Trial. Pediatric Physical Therapy. 2022 Jan 1;34(1):10–5.

266. Aanpreung P, Vanprapar N, Parkpreaw C, Boonyachart C. A Randomized Clinical Trial Comparing the Efficacy of Ranitidine and Famotidine on Intragastric Acidity in Critically Ill Pediatric Patients.

267. Akinci SB, Kanbak M, Guler A, Aypar U. Remifentanil versus fentanyl for short-term analgesia-based sedation in mechanically ventilated postoperative children. Paediatr Anaesth. 2005 Oct;15(10):870–8.

268. Albers MJIJ, Steyerberg EW, Hazebroek FWJ, Mourik M, Borsboom GJJM, Rietveld T, et al. Glutamine supplementation of parenteral nutrition does not improve intestinal permeability, nitrogen balance, or outcome in newborns and infants undergoing digestive-tract surgery: Results from a double-blind, randomized, controlled trial. Ann Surg. 2005 Apr;241(4):599–606.

269. Anene O, Meert KL, Uy H, Simpson P, Sarnaik AP. Ask a Question [Clinical Investigation] Critical Care Medicine Dexamethasone for the prevention of postextubation airway obstruction: A prospective, randomized, double-blind, placebo-controlled trial|. Crit Care Med. 1996;24(10):1666–9.

270. Arino M, Barrington JP, Morrison AL, Gillies D. Management of the changeover of inotrope infusions in children. Intensive Crit Care Nurs. 2004 Oct;20(5):275–80.

271. Arnold JH, Hanson JH, Toro-Figuero LO, Gutierrez J, Berens RJ, Anglin DL. Prospective, randomized comparison of high-frequency oscillatory ventilation and conventional mechanical ventilation in pediatric respiratory failure. Crit Care Med. 1994;22(10):1530–9.

272. Barret JP, Jeschke MG, Herndon DN. Selective decontamination of the digestive tract in severely burned pediatric patients. Burns [Internet]. 2001;27:439–45. Available from: www.elsevier.com/locate/burns

273. Barton P, Garcia J, Kouatli A, Kitchen L, Zorka A, Lindsay C, et al. Hemodynamic Effects of IV Milrinone Lactate in Pediatric Patients With Septic Shock* A Prospective, Double-Blinded, Randomized, Placebo-Controlled, Interventional Study. Chest [Internet]. 1996;109(5):1302–14. Available from: http://journal.publications.chestnet.org/

274. Bindl L, Buderus S, Ramirez M, Kirehhoff P, Lentze MJ, Lentze J, et al. Cisapride reduces postoperative gastrocaecal transit time after cardiac surgery in children. Intensive Care Med. 1996;22:977–80.

275. Briassoulis G, Filippou O, Kanariou M, Hatzis T. Comparative effects of early randomized immune or non-immune-enhancing enteral nutrition on cytokine production in children with septic shock. Intensive Care Med. 2005 Jun;31(6):851–8.

276. Broner CW, Stidham GL, Westenkirchner DF, Watson DC. A prospective, randomized, double-blind comparison of calcium chloride and calcium gluconate therapies for hypocalcemia in critically children ill. Clinical and laboratory observations. 1990;117(6):986–9.

277. Burmester M, Mok Q. Randomised controlled trial comparing cisatracurium and vecuronium infusions in a paediatric intensive care unit. Intensive Care Med. 2005 May;31(5):686–92.

278. Butkovic D, Kralik S, Matolic M, Kralik M, Toljan S, Radesic L. Postoperative analgesia with intravenous fentanyl PCA vs epidural block after thoracoscopic pectus excavatum repair in children. Br J Anaesth. 2007;98(5):677–81.

279. Cai J, Su Z, Shi Z, Zhou Y, Xu Z, Xu Z, et al. Nitric Oxide and Milrinone: Combined Effect on Pulmonary Circulation After Fontan-Type Procedure: A Prospective, Randomized Study. Annals of Thoracic Surgery. 2008 Sep;86(3):882–8.

280. Chiaretti A, Simeone E, Langer A, Butera G, Piastra M, Tortorolo L, et al. Efficacia analgesica del Ketorolac e del Fentanyl in Terapia Intensiva Pediatrica. La Pediatria Medica e Chirurgica. 1997;19:419–24.

281. Cholette JM, Rubenstein JS, Alfieris GM, Powers KS, Eaton M, Lerner NB. Children with single-ventricle physiology do not benefit from higher hemoglobin levels post cavopulmonary connection: Results of a prospective, randomized, controlled trial of a restrictive versus liberal red-cell transfusion strategy. Pediatric Critical Care Medicine. 2011;12(1):39–45.

282. Choong K, Bohn D, Fraser DD, Gaboury I, Hutchison JS, Joffe AR, et al. Vasopressin in pediatric vasodilatory shock: A multicenter randomized controlled trial. Am J Respir Crit Care Med. 2009 Oct 1;180(7):632–9.

283. Chowdhury D, Djamaa K, Parnell VA, McMahon C, Sison CP, Klein I. A prospective randomized clinical study of thyroid hormone treatment after operations for complex congenital heart disease. Journal of Thoracic and Cardiovascular Surgery. 2001 Nov 1;122(5):1023–5.

284. Da Silva P, Paulo C, De Oliveira Iglesias S, De Carvalho W, Santana e Meneses F. Bedside transpyloric tube placement in the pediatric intensive care unit: A modified insufflation air technique. Intensive Care Med. 2002;28(7):943–6.

285. Day RW, Allen EM, Witte MK. A randomized, controlled study of the 1-hour and 24-hour effects of inhaled nitric oxide therapy in children with acute hypoxemic respiratory failure. Chest. 1997;12(5):1324–31.

286. Day RW, Md ;, Guarin M, Lynch JM, Rrt ;, Vernon DD, et al. Inhaled nitric oxide in children with severe lung disease: Results of acute and prolonged therapy with two concentrations. Crit Care Med. 1996;24(2):215–21.

287. De Barbieri I, Frigo AC, Zampieron A. Quick change versus double pump while changing the infusion of inotropes: an experimental study BACKGROUND OF THE STUDY. Nursing in Critical Care . 2009;14(4):200–6.

288. de Moraes MA, Bonatto RC, Carpi MF, Ricchetti SMQ, Padovani CR, Fioretto JR. Comparison between intermittent mandatory ventilation and synchronized intermittent mandatory ventilation with pressure support in children. J Pediatr (Rio J). 2009 Jan;85(1):15–20.

289. De Neef M, Heijboer H, Van Woensel JBM, De Haan RJ. The efficacy of heparinization in prolonging patency of arterial and central venous catheters in children: A randomized double-blind trial. Pediatr Hematol Oncol. 2002 Dec;19(8):553–60.

290. De Oliveira CF, De Oliveira DSF, Gottschald AFC, Moura JDG, Costa GA, Ventura AC, et al. ACCM/PALS haemodynamic support guidelines for paediatric septic shock: An outcomes comparison with and without monitoring central venous oxygen saturation. Intensive Care Med. 2008 Jun;34(6):1065–75.

291. Dobyns EL, Cornfield DN, Anas NG, Fortenberry JD, Tasker RC, Lynch A, et al. Multicenter randomized controlled trial of the effects of inhaled nitric oxide therapy on gas exchange in children with acute hypoxemic respiratory failure. J Pediatr. 1999;134(4):406–12.

292. Eddleston JM, Booker PD, Green JR. Use of ranitidine in children undergoing cardiopulmonary bypass. Crit Care Med. 1989;17(1):26–9.

293. El-Bayoumi MA, El-Refaey AM, Abdelkader AM, El-Assmy MMA, Alwakeel AA, El-Tahan HM. Comparison of intravenous immunoglobulin and plasma exchange in treatment of mechanically ventilated children with Guillain Barré syndrome: A randomized study. Crit Care. 2011 Jul 11;15(4).

294. Fallah R, Gofrani M. Comparison of intravenous lidocaine and midazolam infusion for refractory convulsive status epilepticus in children. Journal of Pediatric Neurology. 2007;5:287–90.

295. Fanconi S, Kloti J, Meuli M, Zaugg H, Zachmann M. Intensive Care Medicine Dexamethasone therapy and endogenous cortisol production in severe pediatric head injury. Intensive Care Med. 1988;14:63–166.

296. Farias J, Retta A, Alía I, Olazarri F, Esteban A, Golubicki A, et al. A comparison of two methods to perform a breathing trial before extubation in pediatric intensive care patients. Intensive Care Med. 2001;27(10):1649–54.

297. Fraisse A, Butrous G, Taylor MB, Oakes M, Dilleen M, Wessel DL. Intravenous sildenafil for postoperative pulmonary hypertension in children with congenital heart disease. Intensive Care Med. 2011 Mar;37(3):502–9.

298. Fram RY, Cree MG, Wolfe RR, Mlcak RP, Qian T, Chinkes DL, et al. Intensive insulin therapy improves insulin sensitivity and mitochondrial function in severely burned children. Crit Care Med. 2010;38(6):1475–83.

299. Gharpure V, Meert KL, Sarnaik AP. Efficacy of erythromycin for postpyloric placement of feeding tubes in critically ill children: A randomized, double-blind, placebo controlled study. Journal of Parenteral and Enteral Nutrition. 2001;25(3):160–5.

300. Gooding AM, Bastian JF, Peterson BM, Wilson NW. Safety and Efficacy of Intravenous Immunoglobulin Prophylaxis in Pediatric Head Trauma Patients: A Double-Blind Controlled Trial. J Crit Care. 1993;8(4):212–6.

301. Gottschlich MM, Jenkins ME, Mayes T, Khoury J, Kagan RJ, Warden GD. An Evaluation of the Safety of Early vs Delayed Enteral Support and Effects on Clinical, Nutritional, and Endocrine Outcomes After Severe Burns. Journal of Burn Care & Rehabilitation [Internet]. 2002;23(6):401–15. Available from: https://academic.oup.com/jbcr/article/23/6/401/4734523

302. Greissman A, Silver P, Nimkoff L, Sagy M. Albumin bolus administration versus continuous infusion in critically ill hypoalbuminemic pediatric patients. Intensive Care Med. 1996;22:495–9.

303. Gupta A, Daggett C, Drant S, Rivero N, Lewis A. Prospective randomized trial of ketorolac after congenital heart surgery. J Cardiothorac Vasc Anesth. 2004;18(4):454–7.

304. Harel Y, Vardi A, Quigley R, Brink LW, Manning SC, Carmody TJ, et al. Extubation failure due to post-extubation stridor is better correlated with neurologic impairment than with upper airway lesions in critically ill pediatric patients. Int J Pediatr Otorhinolaryngol. 1997;39:147–58.

305. Hatem TP, Lira PIC, Mattos SS. The therapeutic effects of music in children following cardiac surgery. J Pediatr (Rio J). 2006;82(3):186–92.

306. Heulitt MJ, Farrington EA, O’Shea TM, Stoltzman SM, Srubar NB, Levin DL. Double-blind, randomized, controlled trial of papaverine-containing infusions to prevent failure of arterial catheters in pediatric patients. Crit Care Med. 1993;21(6):825–9.

307. Honeycutt TCB, El Khashab M, Wardrop RM, McNeal-Trice K, Honeycutt ALB, Christy CG, et al. Probiotic administration and the incidence of nosocomial infection in pediatric intensive care: A randomized placebo-controlled trial. Pediatric Critical Care Medicine. 2007;8(5):452–8.

308. Horn D, Chaboyer W. Gastric Feeding In Critically Ill Children: a Randomized Controlled Trial. American Journal of Critical Care. 2003;12(5):461–8.

309. Hutchison JS, Ward RE, Lacroix J, Hébert PC, Barnes MA, Bohn DJ, et al. Hypothermia Therapy after Traumatic Brain Injury in Children. N Engl J Med [Internet]. 2008;358(23):2447–56. Available from: www.nejm.org

310. Ibrahim TS, El-Mohamady HS. Inhaled nitric oxide and prone position: how far they can improve oxygenation in pediatric patients with acute respiratory distress syndrome? Journal of Medical Sciences. 2007;7(3):390–5.

311. Jaarsma AS, Knoester H, Van Rooyen F, Bos AP, Jaarsma AS. Biphasic positive airway pressure ventilation (PeV+) in children. Crit Care [Internet]. 2001;5(3):174–7. Available from: http://ccforum.com/content/5/3/174

312. Jacobs BR, Barr LL, Brilli RJ, Lyons KA, Wong HR. Intracatheter nitroglycerin infusion fails to prevent catheter-related venous thrombosis: A randomized, controlled trial. Intensive Care Med. 2001;27(1):187–92.

313. Jácomo ADN, Carmona F, Matsuno AK, Manso PH, Carlotti APCP. Effect of Oral Hygiene with 0.12% Chlorhexidine Gluconate on the Incidence of Nosocomial Pneumonia in Children Undergoing Cardiac Surgery. Infect Control Hosp Epidemiol. 2011 Jun;32(6):591–6.

314. Jeschke MG, Finnerty CC, Suman OE, Kulp G, Mlcak RP, Herndon DN. The effect of oxandrolone on the endocrinologic, inflammatory, and hypermetabolic responses during the acute phase postburn. Ann Surg. 2007 Sep;246(3):351–60.

315. Jeschke MG, Norbury WB, Finnerty CC, Branski LK, Herndon DN. Propranolol does not increase inflammation, sepsis, or infectious episodes in severely burned children. Journal of Trauma - Injury, Infection and Critical Care. 2007 Mar;62(3):676–81.

316. Klein SM, Hauser GJ, Anderson BD, Shad AT, Gootenberg JE, Dalton HJ, et al. Comparison of intermittent versus continuous infusion of propofol for elective oncology procedures in children*. Pediatric Critical Care Medicine. 2003;4(1):78–82.

317. Klinge JM, Scharf J, Hofbeck M, Gerling S, Bonakdar S, Singer H. Intermittent administration of furosemide versus continuous infusion in the postoperative management of children following open heart surgery. Intensive Care Med. 1997;23:693–7.

318. Krafte-Jacobs B, Persinger M, Carver J, Moore L, Brilli R. Rapid placement of transpyloric feeding tubes: a comparison of pH-assisted and standard insertion techniques in children. Pediatrics. 1996;98(2).

319. Lacroix J, Hébert PC, Hutchison JS, Hume HA, Tucci M, Ducruet T, et al. Transfusion Strategies for Patients in Pediatric Intensive Care Units. N Engl J Med [Internet]. 2007;356(16):1609–19. Available from: www.nejm.org

320. Lacroix J, Infante-Rivard C, Gauthier M, Rousseau E, van Doesburg N. Upper gastrointestinal tract bleeding acquired in a pediatric intensive care unit: prophylaxis trial with cimetidine. J Pediatr. 1986;108(6):1015–8.

321. Leenen FHH, Balfe JA, Pelech AN, Barker GA, Balfe JW, Olley PM. Postoperative hypertension after repair of coarctation of aorta in children: protective effect of propranolol? Am Heart J. 1987;113(5):1164–73.

322. Lenz AM, Vassallo JC, Moreno GE, Althabe M, Gomez S, Magliola R, et al. Prevention of catheter-related infection: usefulness and cost-effectiveness of antiseptic catheters in children. Arch Argent Pediatr. 2010;108(3):209–15.

323. Levy I, Katz J, Solter E, Samra Z, Vidne B, Birk E, et al. Chlorhexidine-impregnated dressing for prevention of colonization of central venous catheters in infants and children: A randomized controlled study. Pediatric Infectious Disease Journal. 2005;24(8):676–9.

324. Li H, Lu G, Shi W, Zheng S. Protective Effect of Moderate Hypothermia on Severe Traumatic Brain Injury in Children. J Neurotrauma. 2009;25:1905–9.

325. Li ZP, Cao Q, Xing QS. Effect of Milkvetch Injection on Cardiac Function and Hemodynamics in Children with Tetralogy of Fallot after Radical Operation. Chinese Journal of Integrated Traditional and Western Medicine. 2003;23(12):891–4.

326. Lieh-Lai MW, Kauffman R, Uy HG, Danjin M, Simpson PM. Ask a Question [Pediatric Critical Care] Critical Care Medicine A randomized comparison of ketorolac tromethamine and morphine for postoperative analgesia in critically ill children. 1999;27(12):2786–91.

327. Coulthard MG, Long DA, Ullman AJ, Ware RS. A randomised controlled trial of Hartmann’s solution versus half normal saline in postoperative paediatric spinal instrumentation and craniotomy patients. Arch Dis Child. 2012 Jun;97(6):491–6.

328. Lopez-Herce J, Velasco LA, Codoceo R, Dominguez MAD, Jimenez E, Tarrio FR. Ranitidine prophylaxis in acute gastric mucosal damage in critically ill pediatric patients. Crit Care Med. 1988;16(6):591–3.

329. Loukanov T, Bucsenez D, Springer W, Sebening C, Rauch H, Roesch E, et al. Comparison of inhaled nitric oxide with aerosolized iloprost for treatment of pulmonary hypertension in children after cardiopulmonary bypass surgery. Clinical Research in Cardiology. 2011 Jul;100(7):595–602.

330. Lucas Da Silva PS, Oliveira Iglesias SB, Leão FVF, Aguiar VE, Brunow De Carvalho W. Procedural sedation for insertion of central venous catheters in children: Comparison of midazolam/fentanyl with midazolam/ketamine. Paediatr Anaesth. 2007 Apr;17(4):358–63.

331. Luciani GB, Nichani S, Chang AC, Wells WJ, Newth CJ, Starnes VA. Continuous Versus Intermittent Furosemide Infusion in Critically Ill Infants After Open Heart Operations. Ann Thorac Surg [Internet]. 1997;64:1133–9. Available from: http://www.sts.org/annals

332. Lukas JC, Karikas G, Gazouli M, Kalabalikis P, Hatzis T, Macheras P. Pharmacokinetics of Teicoplanin in an ICU Population of Children and Infants. Pharm Res. 2004;21(11):2064–71.

333. Lyons KA, Brilli RJ, Wieman RA, Jacobs BR. Continuation of transpyloric feeding during weaning of mechanical ventilation and tracheal extubation in children: A randomized controlled trial. Journal of Parenteral and Enteral Nutrition. 2002;26(3):209–13.

334. Möller JC, Schaible T, Roll C, Schiffmann JH, Bindl L, Schrod L, et al. Treatment with bovine surfactant in severe acute respiratory distress syndrome in children: A randomized multicenter study. Intensive Care Med. 2003 Mar 1;29(3):437–46.

335. Macnab AJ, Levine M, Glick N, Macready J, Susak L, Elliott M, et al. Midazolam following open heart surgery in children: haemodynamic effects of a loading dose. Paediatr Anaesth. 1996;6:387–97.

336. Marik PE, Havlik I, Monteagudo FSE, Lipman J. The phannacokinetics of amikacin in critically ill adult and paediatric patients: comparison of once-versus twice-daily dosing regimens. Journal of Antimicrobial Chemotherapy [Internet]. 1991;27:81–9. Available from: http://jac.oxfordjournals.org/

337. Marraro GA, Luchetti M, Spada C, Galassini E, Giossi M, Piero AMP. Selective medicated (normal saline and exogenous surfactant) bronchoalveolar lavage in severe aspiration syndrome in children. Pediatric Critical Care Medicine. 2007 Sep;8(5):476–81.

338. Melchers P, Maluck A, Suhr L, Scholten S, Lehmkuhl G. An early onset rehabilitation program for children and adolescents after traumatic brain injury (TBI): methods and first results. Restor Neurol Neurosci. 1999;14:153–60.

339. Miller O, Tang SF, Keech A, Pigott NB, Beller E, Celermajer DS. Inhaled nitric oxide and prevention of pulmonary hypertension after congenital heart surgery: A randomised double-blind study. Lancet. 2000 Oct 28;356(9240):1464–9.

340. Molon M, Piva J, Karcher P, Baldissera T. Clonidine Associated to Morphine and Midazolam in Children Submitted to Mechanical Ventilation: Randomized, Double Blind and Placebo Controlled Study. Rev Bras Ter Intensiva. 2007;19(3):284–91.

341. Montañana PÃ, Modesto I Alapont V, Ocón AP, López PO, López Prats JL, Toledo Parreño JD. The use of isotonic fluid as maintenance therapy prevents iatrogenic hyponatremia in pediatrics: A randomized, controlled open study. Pediatric Critical Care Medicine. 2008;9(6):589–97.

342. Morrow B, Futter M, Argent A. A recruitment manoeuvre performed after endotracheal suction does not increase dynamic compliance in ventilated paediatric patients: a randomised controlled trial. Australian Journal of Physiotherapy. 2007;53:163–9.

343. Nahum E, Levy I, Katz J, Samra Z, Ashkenazi S, Ben-Ari J, et al. Efficacy of subcutaneous tunneling for prevention of bacterial colonization of femoral central venous catheters in critically ill children. Pediatr Infect Dis J. 2002;21(11):1000–4.

344. Namachivayam P, Theilen U, Butt WW, Cooper SM, Penny DJ, Shekerdemian LS. Sildenafil prevents rebound pulmonary hypertension after withdrawal of nitric oxide in children. Am J Respir Crit Care Med. 2006 Nov 1;174(9):1042–7.

345. Nutman J, Brooks LJ, Deakins KM, Baldesare KK, Witte MK, Reed MD. Racemic versus l-epinephrine aerosol in the treatment of postextubation laryngeal edema: results from a prospective, randomized, double-blind study. Crit Care Med. 1994;22(10):1591–4.

346. Parkinson L, Hughes J, Gill A, Billingham I, Ratcliffe J, Choonara I. A randomized controlled trial of sedation in the critically ill. Paediatr Anaesth. 2004;7:405–10.

347. Phipps LM, Thomas NJ, Ginder B, Weber M, Hulse MA. A Randomized Controlled Trial Comparing Three Different Techniques of Nasojejunal Feeding Tube Placement in Critically Ill Children. Crit Care Med. 2002 Dec;30(Supplement):A143.

348. Pierce CM, Wade A, Mok Q. Heparin-bonded central venous lines reduce thrombotic and infective complications in critically ill children Related papers. Intensive Care Med. 2000;26:967–72.

349. Prabhakaran P, Reddy AT, Oakes WJ, King WD, Winkler MK, Givens TG. A pilot trial comparing cerebral perfusion pressure-targeted therapy to intracranial pressure-targeted therapy in children with severe traumatic brain injury KEY WORDS • brain injury • trauma • cerebral edema • Glasgow Coma Scale • Glasgow Outcome Scale • intracranial pressure • cerebral perfusion pressure. J Neurosurg: Pediatrics. 2004;5:454–9.

350. Preutthipan A, Poomthavorn P, Sumanapisan A, Chinrat B, Thasuntia S, Plitponkarnpim A, et al. A Prospective, Randomized Double-blind Study in Children Comparing Two Doses of Nebulized L-epinephrine in Postintubation Croup. J Med Assoc Thai [Internet]. 2005;88(4). Available from: http://www.medassocthai.org/journal

351. Prins SA, Van Dijk M, Van Leeuwen P, Searle S, Anderson BJ, Tibboel D, et al. Pharmacokinetics and analgesic effects of intravenous propacetamol vs rectal paracetamol in children after major craniofacial surgery. Paediatr Anaesth. 2008 Jul;18(7):582–92.

352. Ream RS, Hauver JF, Lynch RE, Kountzman B, Gale GB, Mink RB. Low-dose inhaled nitric oxide improves the oxygenation and ventilation of infants and children with acute, hypoxemic respiratory failure. Crit Care Med [Internet]. 2015;27(5):989–96. Available from: http://ovidsp.tx.ovid.com.proxy.bib.uottawa.calsp-3.17.Oalovidweb.cgi

353. Reeves JH, Butt WW, Shann F, Layton J, Stewart A, Waring P, et al. Continuous plasmafiltration in sepsis syndrome Author Information. Critical Care Medicine Issue [Internet]. 1999;27(10):2096–104. Available from: http://ovidsp.tx.ovid.com.proxy.bib.uottawa.calsp-3.17.0a/ovidweb.cgi

354. Riethmueller J, Borth-Bruhns T, Kumpf M, Vonthein R, Wiskirchen J, Stern M, et al. Recombinant human deoxyribonuclease shortens ventilation time in young, mechanically ventilated children. Pediatr Pulmonol. 2006 Jan;41(1):61–6.

355. Rushforth K. A randomised controlled trial of weaning from mechanical ventilation in Paediatric Intensive Care (PIC). Methodological and practical issues. Intensive Crit Care Nurs. 2005 Apr;21(2):76–86.

356. Ruza F, Alvarado F, Herruzo R, Delgado MA, Garcia S, Dorao P, et al. Prevention of nosocomial infection in a pediatric intensive care unit (PICU) through the use of selective digestive decontamination. Eur J Epidemiol. 1998;14:719–27.

357. Sakellaris G, Kotsiou M, Tamiolaki M, Kalostos G, Tsapaki E, Spanaki M, et al. Prevention of complications related to traumatic brain injury in children and adolescents with creatine administration: An open label randomized pilot study. Journal of Trauma - Injury, Infection and Critical Care. 2006 Aug;61(2):322–9.

358. Samransamruajkit R, Jirapaiboonsuk S, Siritantiwat S, Tungsrijitdee O, Deerojanawong J, Sritippayawan S, et al. Effect of frequency of ventilator circuit changes (3 vs 7 days) on the rate of ventilator-associated pneumonia in PICU. J Crit Care. 2010 Mar;25(1):56–61.

359. Samransamruajkit R, Prapphal N, Deelodegenavong J, Poovorawan Y. Plasma soluble intercellular adhesion molecule-1 (sICAM-1) in pediatric ARDS during high frequency oscillatory ventilation: a predictor of mortality. Asian Pac J Allergy Immunol. 2005;23:181–8.

360. Saul JP, Scott WA, Brown S, Marantz P, Acevedo V, Etheridge SP, et al. Intravenous amiodarone for incessant tachyarrhythmias in children: A randomized, double-blind, antiarrhythmic drug trial. Circulation. 2005 Nov;112(22):3470–7.

361. Schultz TR, Lin RJ, Watzman HM, Durning SM, Hales R, Woodson A, et al. Weaning children from mechanical ventilation: a prospective randomized trial of protocol-directed versus physician-directed weaning. Respir Care. 2001;46(8):772–82.

362. Scoble MK, Copnell B, Taylor A, Kinney S, Shann F. Effect of reusing suction catheters on the occurrence of pneumonia in children. Heart and Lung: Journal of Acute and Critical Care. 2001;30(3):225–33.

363. Semsroth M, Hiesmayr M. Postoperative continuous application of morphine is more effective than bolus application for analgosedation in children. Anaesthesist. 1990;39:552–6.

364. Shahid SK. Efficacy and safety of cefepime in late-onset ventilator-associated pneumonia in infants: A pilot randomized and controlled study. Ann Trop Med Parasitol. 2008 Jan;102(1):63–71.

365. Simakachorn N, Bibiloni R, Yimyaem P, Tongpenyai Y, Varavithaya W, Grathwohl D, et al. Tolerance, safety, and effect on the faecal microbiota of an enteral formula supplemented with pre- and probiotics in critically ill children. J Pediatr Gastroenterol Nutr. 2011 Aug;53(2):174–81.

366. Singh NC, Kissoon N, Al Mofada S, Bennett M, Bohn DJ. Comparison of continuous versus intermittent furosemide administration in postoperative pediatric cardiac patients. Crit Care Med. 1992;20(1):17–21.

367. Singhi S, Järvinen A, Peltola H. Increase in serum osmolality is possible mechanism for the beneficial effects of glycerol in childhood bacterial meningitis. Pediatric Infectious Disease Journal. 2008;27(10):892–6.

368. Singhi S, Murthy A, Singhi P, Jayashree ; M. Continuous Midazolam Versus Diazepam Infusion for Refractory Convulsive Status Epilepticus. J Child Neurol. 2002;17(2):106–10.

369. Sinha A, Jayashree M, Singhi S. Aerosolized L-epinephrine vs budesonide for post extubation stridor: a randomized controlled trial. Indian Pediatr. 2010;47:317–22.

370. Slota M, Green M, Farley A, Janosky J, Carcillo J. The role of gown and glove isolation and strict handwashing in the reduction of nosocomial infection in children with solid organ transplantation. Pediatric Critical Care. 2001;29(2):405–12.

371. Smith SD, Jackson RJ, Hannakan CJ, Wadowsky RM, Tzakis AG, Rowe MI. Selective decontamination in pediatric liver transplants. A randomized prospective study. Transplantation. 1993;55:1306–9.

372. Soler M, Raszynski A, Kandrotas RJ, Sussmane JB, Aznavorian R, Wolfsdorf J. Fewer Interventions in the Immediate Post-Extubation Management of Pediatric Intensive Care Unit Patients: Safety and Cost Containment. J Crit Care. 1997;12(4):173–6.

373. Srinivasan R, Meyer R, Padmanabhan R, Britto J. Clinical Safety of Lactobacillus casei shirota as a Probiotic in Critically Ill Children. J Pediatr Gastroenterol Nutr. 2006;42(2):171–3.

374. Stocker C, Penny DJ, Brizard CP, Cochrane AD, Soto R, Shekerdemian LS. Intravenous sildenafil and inhaled nitric oxide: A randomised trial in infants after cardiac surgery. Intensive Care Med. 2003 Nov;29(11):1996–2003.

375. Tan L li, Huang J fan, Wang H. Effect of rehabilitation training on postoperative recovery of children with congenital heart disease. Chinese Journal of Nursing. 1996;31(6):314–5.

376. Tellez DW, Galvis AG, Storgion SA, Amer HN, Hoseyni M, Deakers TW. Dexamethasone in the prevention of postextubation stridor in children. J Pediatr. 1991;118(2):289–94.

377. Tibballs J, Shann FA, Landau LI. Placebo-controlled trial of prednisolone in children intubated for croup. Lancet. 1992;340:745–8.

378. Umenai T, Shime N, Hashimoto S. Hyperventilation versus standard ventilation for infants in postoperative care for congenital heart defects with pulmonary hypertension. J Anesth. 2009;23(1):80–6.

379. Upadhyay P, Tripathi V, Singh R, Sachan D. Role of hypertonic saline and mannitol in the management of raised intracranial pressure in children: A randomized comparative study. From: Journal of Pediatric Neurosciences. 2010;5(1).

380. Van den Berghe G, de Zegher F, Lauwers P. Dopamine suppresses pituitary function in infants and children. Crit Care Med. 1994;22(11):1747–53.

381. Van Dijk M, Bouwmeester NJ, Duivenvoorden HJ, Koot HM, Tibboel D, Passchier J, et al. Efficacy of continuous versus intermittent morphine administration after major surgery in 0-3-year-old infants; a double-blind randomized controlled trial. Pain [Internet]. 2002;98:305–13. Available from: www.elsevier.com/locate/pain

382. Vlasselaers D, Milants I, Desmet L. Intensive insulin therapy for patients in paediatric intensive care: a prospective, randomised controlled study. The Lancet [Internet]. 2009;373:547–56. Available from: www.thelancet.com

383. Yañez LJ, Yunge M, Emilfork M, Lapadula M, Alcántara A, Fernández C, et al. A prospective, randomized, controlled trial of noninvasive ventilation in pediatric acute respiratory failure. Pediatric Critical Care Medicine. 2008;9(5):484–9.

384. Yildizdas D, Yapicioglu H, Celik U, Sertdemir Y, Alhan E. Terlipressin as a rescue therapy for catecholamine-resistant septic shock in children. Intensive Care Med. 2008 Mar;34(3):511–7.

385. Yildizdas D, Yapýcýoðlu H, Tümgör G, Erbey F, Üniversitesi Týp Fakültesi Ç, Doçenti P, et al. Does polyclonal intravenous immunoglobulin reduce mortality in septic children in the pediatric intensive care unit? Çocuk Saðlýðý ve Hastalýklarý Dergisi. 2005;48:136–41.

386. Yildizdas D, Yapicioglu H, Yilmaz HL. Occurrence of Ventilator-Associated Pneumonia in Mechanically Ventilated Pediatric Intensive Care Patients During Stress Ulcer Prophylaxis With Sucralfate, Ranitidine, and Omeprazole. J Crit Care. 2002;17(4):240–5.

387. Yu K ye, Huang X hua, Li H da. Clinical observation on treatment of gastrointestinal dysfunction by fu’an liquid for retention enema in children with critical illness. Chinese Journal of Integrated Traditional Chinese and Western Medicine. 2002;22(4):261–3.

388. Yung M, Keeley S. Randomised controlled trial of intravenous maintenance fluids. J Paediatr Child Health. 2009 Jan;45(1–2):9–14.

389. Zhang Y qian, Huang B, Liu J hua, Tian L yuan, Hu H fu. Efficacy of ciprofloxacin in the treatment of pediatric severe infections. Chinese Journal of Antibiotics. 2005;7(30):416–9.

390. Zhao K, Wang W, Zhang J, Zhao R, Chen T, Su J, et al. Effects of high-dose mucosolvin on lung functions in infant patients with cardiopulmonary bypass. Heart Surgery Forum. 2011 Aug;14(4).

391. Zobel G, Kuttnig Ma, Grubbauer HM, Semmelrock HJ, Thiel W. Reduction of colonization and infection rate during pediatric intensive care by selective decontamination of the digestive tract. Crit Care Med. 1991;19(10):1242–6.

392. Abdallah I, Shawky H. Milrinone vs. epinephrine in Fallot’s Tetralogy 323. Eg J Anaesth. 2003;19:323–9.

393. Abdollahi T, Sabzevari A, Khakshour A, Nakhaie AA, Sezavar M, Etezadie T, et al. Effect of gastric acid suppressant prophylaxis on incidence of gastrointestinal bleeding in pediatric intensive care unit. Int J Pediatr. 2016;4(11):3917–24.

394. Abraham S, Rameshkumar R, Chidambaram M, Soundravally R, Subramani S, Bhowmick R, et al. Trial of Furosemide to Prevent Acute Kidney Injury in Critically Ill Children: A Double-Blind, Randomized, Controlled Trial. Indian J Pediatr. 2021 Nov 1;88(11):1099–106.

395. Agus MSD, Steil GM, Wypij D, Costello JM, Laussen PC, Langer M, et al. Tight Glycemic Control versus Standard Care after Pediatric Cardiac Surgery. New England Journal of Medicine. 2012 Sep 27;367(13):1208–19.

396. Ahmadinejad M, Hashemian M, Noradini Z, Ahmadipour M. Evaluation the Effect of New Transdermal Oxygen Therapy Method on Healing of Pressure Ulcer in Children. International Journal of Pharmaceutical and Phytopharmacological Research [Internet]. 2020;10(1):61–9. Available from: www.eijppr.com

397. Akyildiz B, Tekerek NU, Pamukcu O, Dursun A, Karakukcu M, Narin N, et al. Comprehensive analysis of liberal and restrictive transfusion strategies in pediatric intensive care unit. J Trop Pediatr. 2018 Apr 1;64(2):118–25.

398. Akyıldız B, Öztürk S, Ülgen-Tekerek N, Doğanay S, Görkem SB. Comparison between high-flow nasal oxygen cannula and conventional oxygen therapy after extubation in pediatric intensive care unit. Turkish Journal of Pediatrics. 2018;60(2):126–33.

399. Almeida HI, Mascarenhas MI, Loureiro HC, Abadesso CS, Nunes PS, Moniz MS, et al. The effect of NaCl 0.9% and NaCl 0.45% on sodium, chloride, and acid-base balance in a PICU population. J Pediatr (Rio J). 2015 Sep 1;91(5):499–505.

400. Amanullah MM, Hamid M, Hanif HM, Muzaffar M, Siddiqui MT, Adhi F, et al. Effect of steroids on inflammatory markers and clinical parameters in congenital open heart surgery: A randomised controlled trial. Cardiol Young. 2016 Mar 1;26(3):506–15.

401. Amrousy D El, Elshehaby W, Feky W El, Elshmaa NS. Safety and Efficacy of Prophylactic Amiodarone in Preventing Early Junctional Ectopic Tachycardia (JET) in Children After Cardiac Surgery and Determination of Its Risk Factor. Pediatr Cardiol. 2016 Apr 1;37(4):734–9.

402. El Amrousy DM, Elshmaa NS, El-Kashlan M, Hassan S, Elsanosy M, Hablas N, et al. Efficacy of prophylactic dexmedetomidine in preventing postoperative junctional ectopic tachycardia after pediatric cardiac surgery. J Am Heart Assoc. 2017;6(3).

403. Anantasit N, Cheeptinnakorntaworn P, Khositseth A, Lertbunrian R, Chantra M. Ultrasound Versus Traditional Palpation to Guide Radial Artery Cannulation in Critically Ill Children: A Randomized Trial: A. Journal of Ultrasound in Medicine. 2017 Dec 1;36(12):2495–501.

404. Angurana SK, Bansal A, Singhi S, Aggarwal R, Jayashree M, Salaria M, et al. Evaluation of effect of probiotics on cytokine levels in critically Ill children with severe sepsis: A double-blind, placebo-controlled trial. Crit Care Med. 2018;46(10):1656–64.

405. Anton N, Cox PN, Massicotte MP, Chait P, Yasui Y, Dinyari PM, et al. Heparin-bonded central venous catheters do not reduce thrombosis in infants with congenital heart disease: A blinded randomized, controlled trial. Pediatrics. 2009 Mar;123(3).

406. babaie sharareh, torki A, Keivanfar M. Efficacy of fentanyl on pain relief during tracheal suctioning in ventilated children: A randomized clinical trial. Trends in Anaesthesia and Critical Care. 2022 Feb 1;42:14–9.

407. Bafaqih H, Almohaimeed S, Thabet F, Alhejaili A, Alarabi R, Zolaly M, et al. Utility of daily routine portable chest X-ray in mechanically ventilated patients in the pediatric intensive care unit. J Pediatr Intensive Care. 2015 Jul 28;03(01):029–34.

408. Barnwal NK, Umbarkar SR, Sarkar M, Dias R. Randomized comparative study of intravenous infusion of three different fixed doses of milrinone in pediatric patients with pulmonary hypertension undergoing open heart surgery. Ann Card Anaesth. 2017 Jul 1;20(3):318–22.

409. Bharathi K, Bhat A, Pruthi G, Simha P. Randomized control study of nebulized colistin as an adjunctive therapy in ventilator-Associated pneumonia in pediatric postoperative cardiac surgical population. Ann Card Anaesth. 2022 Oct 1;25(4):435–40.

410. Bigelow AM, Ghanayem NS, Thompson NE, Scott JP, Cassidy LD, Woods KJ, et al. Safety and Efficacy of Vasopressin After Fontan Completion: A Randomized Pilot Study. Annals of Thoracic Surgery [Internet]. 2019;108:1865–74. Available from: https://doi.org/10.1016/j.athoracsur.2019.06.053

411. Bilan N, Ganji S. Weaning from Ventilator and Effect of Blender-Humidifier on Outcome. Int J Pediatr [Internet]. 2014;2(11):4–6. Available from: http://ijp.mums.ac.ir

412. Bilan N, Ganji S. Comparison of CPAP with Humidifier, Blender, and T-piece on the Outcome of Weaning in Patients with Neurological Disorders. Vol. 9, Patients with Neurological Disorders. Iran J Child Neurol. Spring. 2015.

413. Blackwood B, Tume LN, Morris KP, Clarke M, McDowell C, Hemming K, et al. Effect of a Sedation and Ventilator Liberation Protocol vs Usual Care on Duration of Invasive Mechanical Ventilation in Pediatric Intensive Care Units: A Randomized Clinical Trial. JAMA - Journal of the American Medical Association. 2021 Aug 3;326(5):401–10.

414. Blackwood B, Morris KP, Jordan J, McIlmurray L, Agus A, Boyle R, et al. Co-ordinated multidisciplinary intervention to reduce time to successful extubation for children on mechanical ventilation: The SANDWICH cluster stepped-wedge RCT. Health Technol Assess (Rockv). 2022;26(18):VII–95.

415. Branco RG, Garcia PCR, Piva JP, Conrado GS, Cabral F, Korb C, et al. Pilot Mechanistic Study of Insulin Modulation of Somatotrophic Hormones, Inflammation, and Lipid Metabolism During Critical Illness in Children. Pediatric Critical Care Medicine. 2016 Jan 1;18(1):e35–41.

416. Butragueño-Laiseca L, Manrique Martín G, González Cortés R, Rey Galán C, Martínez de Compañón Martínez de Marigorta Z, Gil Antón J, et al. Multicenter randomized clinical trial comparing dexamethasone versus placebo in preventing upper airway obstruction after extubation in critically ill children. Sci Rep. 2022 Dec 1;12(1).

417. Cavigelli-Brunner A, Hug MI, Dave H, Baenziger O, Buerki C, Bettex D, et al. Prevention of low cardiac output syndrome after pediatric cardiac surgery: A double-blind randomized clinical pilot study comparing dobutamine and milrinone. Pediatric Critical Care Medicine. 2018;19(7):619–25.

418. Ceelie I, De Wildt SN, Van Dijk M, Van Den Berg MMJ, Van Den Bosch GE, Duivenvoorden HJ, et al. Effect of Intravenous Paracetamol on Postoperative Morphine Requirements in Neonates and Infants Undergoing Major Noncardiac Surgery A Randomized Controlled Trial. Journal of American Medical Association [Internet]. 2013;309(2):149–54. Available from: http://jama.jamanetwork.com/

419. Chand R, Roy Chowdhury S, Rupert E, Mandal CK, Narayan P. Benefits of Using High-Volume–Low-Pressure Tracheal Tube in Children Undergoing Congenital Cardiac Surgery: Evidence From a Prospective Randomized Study. Semin Cardiothorac Vasc Anesth. 2018 Sep 1;22(3):300–5.

420. Chi CY, Khanh TH, Thoa LPK, Tseng FC, Wang SM, Thinh LQ, et al. Milrinone therapy for enterovirus 71-induced pulmonary edema and/or neurogenic shock in children: A randomized controlled trial. Crit Care Med. 2013 Jul;41(7):1754–60.

421. Cholette JM, Henrichs KF, Alfieris GM, Powers KS, Phipps R, Spinelli SL, et al. Washing red blood cells and platelets transfused in cardiac surgery reduces postoperative inflammation and number of transfusions: Results of a prospective, randomized, controlled clinical trial. Pediatric Critical Care Medicine. 2012 May;13(3):290–9.

422. Cholette JM, Powers KS, Alfieris GM, Angona R, Henrichs KF, Masel D, et al. Transfusion of cell saver salvaged blood in neonates and infants undergoing open heart surgery significantly reduces RBC and coagulant product transfusions and donor exposures: Results of a prospective, randomized, clinical trial. Pediatric Critical Care Medicine. 2013 Feb;14(2):137–47.

423. Cholette JM, Swartz MF, Rubenstein J, Henrichs KF, Wang H, Powers KS, et al. Outcomes Using a Conservative Versus Liberal Red Blood Cell Transfusion Strategy in Infants Requiring Cardiac Operation. Annals of Thoracic Surgery [Internet]. 2017;103:206–15. Available from: http://dx.doi.org/10.1016/j.athoracsur.2016.05.049

424. Choong K, Arora S, Cheng J, Farrokhyar F, Reddy D, Thabane L, et al. Hypotonic versus isotonic maintenance fluids after surgery for children: A randomized controlled trial. Pediatrics. 2011 Nov;128(5):857–66.

425. Choong K, Awladthani S, Khawaji A, Clark H, Borhan A, Cheng J, et al. Early Exercise in Critically Ill Youth and Children, a Preliminary Evaluation: The wEECYCLE Pilot Trial. Pediatric Critical Care Medicine. 2017 Nov 1;18(11):E546–54.

426. Cox EG, Knoderer CA, Jennings A, Brown JW, Rodefeld MD, Walker SG, et al. A randomized, controlled trial of catheter-related infectious event rates using antibiotic-impregnated catheters versus conventional catheters in pediatric cardiovascular surgery patients. J Pediatric Infect Dis Soc. 2013;2(1):67–70.

427. Da Silva PSL, Fonseca MCMH, Iglesias SBO, Junior EL, De Aguiar VE, De Carvalho WB. Nebulized 0.5, 2.5 and 5 ml l-epinephrine for post-extubation stridor in children: A prospective, randomized, double-blind clinical trial. Intensive Care Med. 2012 Feb;38(2):286–93.

428. da Silva PSL, Reis ME, de Aguiar VE, Fonseca MCM. Use of fentanyl and midazolam in mechanically ventilated children-Does the method of infusion matter? J Crit Care. 2016 Apr 1;32:108–13.

429. Dalili M, Vesal A, Tabib A, Khani-Tafti L, Hosseini S, Totonchi Z. Single dose corticosteroid therapy after surgical repair of fallot’s tetralogy; A randomized controlled clinical trial. Res Cardiovasc Med. 2015;4(1):7.

430. de Carvalho HT, Fioretto JR, Bonatto RC, Ribeiro CF, Martin JG, Carpi MF. Use of Dexamethasone to Prevent Extubation Failure in Pediatric Intensive Care Unit: A Randomized Controlled Clinical Trial. J Pediatr Intensive Care. 2022 Mar;11(01):041–7.

431. de Gast-Bakker DH, de Wilde RBP, Hazekamp MG, Sojak V, Zwaginga JJ, Wolterbeek R, et al. Safety and effects of two red blood cell transfusion strategies in pediatric cardiac surgery patients: a randomized controlled trial. Intensive Care Med. 2013 Nov 1;39(11):2011–9.

432. de Jong M, Lucas C, Bredero H, van Adrichem L, Tibboel D, van Dijk M. Does postoperative “M” technique® massage with or without mandarin oil reduce infants’ distress after major craniofacial surgery? J Adv Nurs. 2012 Aug;68(8):1748–57.

433. De Kleijn ED, De Groot R, Hack CE, Mulder PGH, Engl W, Moritz B, et al. Activation of protein C following infusion of protein C concentrate in children with severe meningococcal sepsis and purpura fulminans: A randomized, double-blinded, placebo-controlled, dose-finding study. Crit Care Med. 2003 Jun 1;31(6):1839–47.

434. De Souza TH, Brandão MB, Santos TM, Pereira RM, Nogueira RJN. Ultrasound guidance for internal jugular vein cannulation in PICU: A randomised controlled trial. Arch Dis Child. 2018 Oct 1;103(10):952–6.

435. Derkx B, Wittes J, Mccloskey R. Randomized, Placebo-Controlled Trial of HA-1A, a Human Monoclonal Antibody to Endotoxin, in Children with Meningococcal Septic Shock European Pediatric Meningococcal Septic Shock Trial. Clinical Infectious Diseases. 1999;28:770–7.

436. Dong MF, Ma ZS, Wang JT, Chai SD, Tang PZ, Wang LX. Effect of Preoperational Mechanical Ventilation on Short-Term Postoperative Outcomes in Patients with Severe Tetralogy of Fallot. Heart Lung Circ. 2012 Nov;21(11):679–83.

437. Duffett M, Choong K, Foster J, Cheng J, Meade MO, Menon K, et al. Clonidine in the sedation of mechanically ventilated children: A pilot randomized trial. J Crit Care. 2014;29(5):758–63.

438. Duyu M, Karakaya Z, Yazici P, Yavuz S, Yersel NM, Tascilar MO, et al. Comparison of chlorhexidine impregnated dressing and standard dressing for the prevention of central-line associated blood stream infection and colonization in critically ill pediatric patients: A randomized controlled trial. Pediatrics International. 2022 Jan 1;64(1).

439. Düzkaya DS, Yildiz S. Effect of two different feeding methods on preventing ventilator associated pneumonia in the paediatric intensive care unit (PICU): A randomised controlled study. Australian Critical Care. 2016 Aug 1;29(3):139–45.

440. Düzkaya DS, Sahiner NC, Uysal G, Yakut T, Çitak A. Chlorhexidine-impregnated dressings and prevention of catheter-associated bloodstream infections in a pediatric intensive care unit. Crit Care Nurse. 2016;36(6):e1–7.

441. Düzkaya DS, Uysal G, Bozkurt G, Yakut T, Çitak A. Povidone-Iodine, 0.05% chlorhexidine gluconate, or water for periurethral cleaning before indwelling urinary catheterization in a pediatric intensive care: A randomized controlled trial. Journal of Wound, Ostomy and Continence Nursing. 2017;44(1):84–8.

442. Edwards JD, Williams EP, Wagman EK, McHale BL, Malone CT, Kernie SG. A Single-Centered Randomized Controlled Trial of Primary Pediatric Intensivists and Nurses. J Intensive Care Med. 2022 Dec 1;37(12):1580–6.

443. El-beleidy ASE din, Khattab AAEH, El-Sherbini SA, Al-gebaly HF. Automatic Tube Compensation versus Pressure Support Ventilation and Extubation Outcome in Children: A Randomized Controlled Study. ISRN Pediatr. 2013 Feb 26;2013:1–6.

444. El-Nawawy A, Khater D, Omar H, Wali Y. Evaluation of early corticosteroid therapy in management of pediatric septic shock in pediatric intensive care patients: A randomized clinical study. Pediatric Infectious Disease Journal. 2017;36(2):155–9.

445. El-Nawawy AA, Omar OM, Khalil M. Intraosseous versus intravenous access in pediatric septic shock patients admitted to Alexandria University pediatric intensive care unit. J Trop Pediatr. 2018 Apr 1;64(2):132–40.

446. El-Nawawy AA, Elshinawy MI, Khater DM, Moustafa AA, Hassanein NM, Wali YA, et al. Outcome of Early Hemostatic Intervention in Children With Sepsis and Nonovert Disseminated Intravascular Coagulation Admitted to PICU: A Randomized Controlled Trial. Pediatric Critical Care Medicine. 2021 Mar 1;22(3):E168–77.

447. Elshinawy M, Kamal M, Nazir H, Khater D, Hassan R, Elkinany H, et al. Sepsis-related anemia in a pediatric intensive care unit: transfusion-associated outcomes. Transfusion (Paris). 2020 Feb 1;60(S1):S4–9.

448. Erdoğan Ç, Turan T, Pınar B. The effect of maternal voice for procedural pain in paediatric intensive care unit: A randomised controlled trial. Intensive Crit Care Nurs. 2020 Feb 1;56.

449. Erickson SJ, Millar J, Anderson BJ, Festa MS, Straney L, Shehabi Y, et al. Dexmedetomidine Sedation in Mechanically Ventilated Critically Ill Children: A Pilot Randomized Controlled Trial. Pediatric Critical Care Medicine. 2020 Sep 1;21(9):E731–9.

450. Fatehi S, Eshaghi H, Sharifzadeh M, Mirrahimi B, Qorbani M, Tanzifi P, et al. A randomized clinical trial evaluating the efficacy of colistin loading dose in critically ill children. J Res Pharm Pract. 2019;8(4):196.

451. Fayazi S, Adineh M, Fard SZ, Payam HF, Batvandy ZA. Comparing Two Methods of Enteral Nutrition in Terms of their Complications and the Time Needed to Reach Goal Calorie in Children Hospitalized in ICU [Internet]. Vol. 4, Int J Pediatr. 2016. Available from: http://ijp.mums.ac.ir

452. Fayyazi A, Karimzadeh P, Torabian S, Damadi S, Khaje A. Comparison of intravenous midazolam drip with intermittent intravenous diazepam in the treatment of refractory serial seizures in children. Iran J Child Neurol. 2012;6(3):15–9.

453. Fink EL, Beers SR, Houtrow AJ, Richichi R, Burns C, Doughty L, et al. Early Protocolized Versus Usual Care Rehabilitation for Pediatric Neurocritical Care Patients: A Randomized Controlled Trial. Pediatric Critical Care Medicine. 2019 Jun 1;20(6):540–50.

454. Fioretto JR, Ribeiro CF, Carpi MF, Bonatto RC, Moraes MA, Fioretto EB, et al. Comparison between noninvasive mechanical ventilation and standard oxygen therapy in children up to 3 years old with respiratory failure after extubation: A pilot prospective randomized clinical study. Pediatric Critical Care Medicine. 2015 Feb 13;16(2):124–30.

455. Foronda FK, Troster EJ, Farias JA, Barbas CS, Ferraro AA, Faria LS, et al. The impact of daily evaluation and spontaneous breathing test on the duration of pediatric mechanical ventilation: A randomized controlled trial. Crit Care Med. 2011;39(11):2526–33.

456. Garcia Guerra G, Joffe AR, Sheppard C, Hewson K, Dinu IA, Hajihosseini M, et al. Music Use for Sedation in Critically ill Children (MUSiCC trial): a pilot randomized controlled trial. J Intensive Care. 2021 Dec 1;9(1).

457. Ghasemzadeh B, Azizi B, Azemati S, Bagherinasab M. The Effects of Dexmedetomidine Prescription in Paediatric Patients With Pulmonary Hypertension Under Congenital Heart Surgery. Acta Med Iran. 2020;58(4):171–6.

458. Gilbert RE, Mok Q, Dwan K, Harron K, Moitt T, Millar M, et al. Impregnated central venous catheters for prevention of bloodstream infection in children (the CATCH trial): A randomised controlled trial. The Lancet. 2016 Apr 23;387(10029):1732–42.

459. Guerra GG, Joffe AR, Seal R, Phillipos E, Wong M, Moez EK, et al. Pilot randomized controlled trial on early and late remote ischemic preconditioning prior to complex cardiac surgery in young infants. Paediatr Anaesth. 2017 Apr 1;27(4):433–41.

460. Guitart C, Rodríguez-Fanjul J, Bobillo-Perez S, Carrasco JL, Inarejos Clemente EJ, Cambra FJ, et al. An algorithm combining procalcitonin and lung ultrasound improves the diagnosis of bacterial pneumonia in critically ill children: The PROLUSP study, a randomized clinical trial. Pediatr Pulmonol. 2022 Mar 1;57(3):711–23.

461. Gupta K, Gupta VK, Muralindharan J, Singhi S. Randomized controlled trial of interrupted versus continuous sedative infusions in ventilated children. Pediatric Critical Care Medicine. 2012 Mar;13(2):131–5.

462. Habibzadeh M, Shafa A, Zamani H. The Use of Albumin Add-on Therapy to Loop Diuretic for the Management of Pleural Effusion in Mechanically Ventilated Ill Children. Journal of Kerman University of Medical Sciences. 2021 Nov 1;28(6):568–74.

463. Han L, Feng R, Meng J. Treatment of severe pediatric pneumonia by antibiotic de-escalation therapy. Int J Clin Exp Med [Internet]. 2018;11(3):2610–6. Available from: www.ijcem.com/

464. Handral A, Veerappa B, Gowda V, Shivappa S, Benakappa N, Benakappa A. Levetiracetam versus fosphenytoin in pediatric convulsive status epilepticus: A randomized controlled trial. J Pediatr Neurosci. 2020;15(3):252.

465. Huang YL, Lei YQ, Xie WP, Cao H, Yu XR, Chen Q. Effect of music therapy on infants who underwent mechanical ventilation after cardiac surgery. J Card Surg. 2021 Dec 1;36(12):4460–4.

466. Hünseler C, Balling G, Röhlig C, Blickheuser R, Trieschmann U, Lieser U, et al. Continuous infusion of clonidine in ventilated newborns and infants: A randomized controlled trial. Pediatric Critical Care Medicine. 2014;15(6):511–22.

467. Hussein R. Randomized Prospective Double Blinded Comparative Study Between Dexmedetomidine and Fentanyl for Sedation in Children in Post-Operative Paediatric Surgical Intensive Care Unit. Asian Academy of Management Journal. 2013;11(4).

468. Ibrahim H, Mansour M, El Gendy YG. Peptide-based formula versus standard-based polymeric formula for critically ill children: Is it superior for patients’ tolerance? Archives of Medical Science. 2020;16(2):592–6.

469. Ilić MK, Madžarac G, Babić I, Milavić B. Lavage with diluted surfactant as a treatment option for atelectasis in pediatric intensive care patients. Paediatria Croatica. 2015 Jan 1;59(1):32–8.

470. Ingelse SA, Geukers VG, Dijsselhof ME, Lemson J, Bem RA, van Woensel JB. Less Is More?—A Feasibility Study of Fluid Strategy in Critically Ill Children With Acute Respiratory Tract Infection. Front Pediatr. 2019 Dec 10;7.

471. Isildak FU, Yavuz Y. Comparison of Del Nido and Blood Cardioplegia in Pediatric Patients Undergoing Surgical Repair for Congenital Heart Disease. Pediatr Cardiol. 2021 Aug 1;42(6):1388–93.

472. J5 Study Group. Treatment of Severe Infectious Purpura in Children with Human Plasma from Donors Immunized with Escherichia coli J5: A Prospective Double-Blind Study. J Infect Dis. 1992;165:695–701.

473. Jack T, Boehne M, Brent BE, Hoy L, Köditz H, Wessel A, et al. In-line filtration reduces severe complications and length of stay on pediatric intensive care unit: A prospective, randomized, controlled trial. Intensive Care Med. 2012 Jun;38(6):1008–16.

474. Jalil Y, Damiani F, Astudillo C, Villarroel G, Barañao P, Bustos E, et al. Impact of a noninvasive ventilation protocol in hospitalized children with acute respiratory failure. Respir Care. 2017 Dec 1;62(12):1533–9.

475. Jin G, Guo X, Abduhar A, Qu M, Wei N, Zhang L. THE EARLY INTERVENTION STRATEGY OF THE COMBINATION OF TRADITIONAL CHINESE AND WESTERN MEDICINE IN THE TREATMENT OF PICU PNEUMONIA BY A VENTILATOR. Acta Medica Mediterranea. 2022;38(4):2651–4.

476. Jordan I, Balaguer M, Esteban ME, Cambra FJ, Felipe A, Hernández L, et al. Glutamine effects on heat shock protein 70 and interleukines 6 and 10: Randomized trial of glutamine supplementation versus standard parenteral nutrition in critically ill children. Clinical Nutrition. 2015 Feb 1;35(1):34–40.

477. Jorro Barón FA, Meregalli CN, Rombolá VA, Bolasell C, Pigliapoco VE, Bartoletti SE, et al. Hypotonic versus isotonic intravenous maintenance fluids in critically ill pediatric patients: a randomized clinical trial. Arch Argent Pediatr. 2013 Jul;111(4):281–7.

478. Jouvet PA, Payen V, Gauvin F, Emeriaud G, Lacroix J. Weaning children from mechanical ventilation with a computer-driven protocol: A pilot trial. Intensive Care Med. 2013 May;39(5):919–25.

479. Kadam S, Tailor K, Kulkarni S, Mohanty S, Joshi P, Rao S. Effect of dexmeditomidine on postoperative junctional ectopic tachycardia after complete surgical repair of tetralogy of Fallot: A prospective randomized controlled study. Ann Card Anaesth. 2015 Jul 1;18(3):323–8.

480. Kaplan JM, Zingarelli B, Krallman K, Tang Girdwood S, Lagory D, Mizuno T, et al. Phase 1 safety and pharmacokinetic study on the use of pioglitazone in critically ill patients with sepsis: a randomized clinical trial. Intensive Care Med. 2018 Nov 1;44(11):2006–8.

481. Karacaer F, Biricik E, Ilgınel M, Tunay D, Topçuoğlu Ş, Ünlügenç H. Bilateral erector spinae plane blocks in children undergoing cardiac surgery: A randomized, controlled study. J Clin Anesth. 2022 Sep 1;80.

482. Ketsuwan S, Tanpowpong P, Ruangwattanapaisarn N, Phaopant S, Suppalarkbunlue N, Kooanantkul C, et al. Intravenous Metoclopramide to Improve the Success Rate of Blind Bedside Post-pyloric Placement of Feeding Tube in Critically Ill Children: A Randomized, Double-Blind, Placebo-Controlled Study. Front Pediatr. 2021 Dec 22;9.

483. Kirbas A, Yalcin Y, Tanrikulu N, Gürer O, Isik Ö. Comparison of inhaled nitric oxide and aerosolized iloprost in pulmonary hypertension in children with congenital heart surgery. Cardiol J. 2012;19(4):387–94.

484. Kishore R, Jhamb U. Effect of protocolized weaning and spontaneous breathing trial vs conventional weaning on duration of mechanical ventilation: A randomized controlled trial. Indian Journal of Critical Care Medicine. 2021 Sep 1;25(9):1059–65.

485. Konuk Sener D, Aydin M, Cangur S, Guven E. The Effect of Oral Care with Chlorhexidine, Vitamin E and Honey on Mucositis in Pediatric Intensive Care Patients: A Randomized Controlled Trial. J Pediatr Nurs. 2019 Mar 1;45:e95–101.

486. Kumar S, Bansal A, Chakrabarti A, Singhi S. Evaluation of efficacy of probiotics in prevention of Candida colonization in a PICU-a randomized controlled trial. Crit Care Med. 2013 Feb;41(2):565–72.

487. Kumar V, Angurana SK, Baranwal AK, Nallasamy K. Nasotracheal vs. Orotracheal Intubation and Post-extubation Airway Obstruction in Critically Ill Children: An Open-Label Randomized Controlled Trial. Front Pediatr. 2021 Sep 16;9.

488. Landrigan CP, Rahman SA, Sullivan JP, Vittinghoff E, Barger LK, Sanderson AL, et al. Effect on Patient Safety of a Resident Physician Schedule without 24-Hour Shifts. New England Journal of Medicine. 2020 Jun 25;382(26):2514–23.

489. Lazarev V V., Sulaimanova ZD, Tsypin LE, Brusov GP, Eryasheva T V. Choice of drug for intravenous fluid therapy in the early postoperative period in children. Obshchaya Reanimatologiya. 2020;16(5):30–6.

490. Lechner E, Hofer A, Leitner-Peneder G, Freynschlag R, Mair R, Weinzettel R, et al. Levosimendan versus milrinone in neonates and infants after corrective open-heart surgery: A pilot study. Pediatric Critical Care Medicine. 2012 Sep;13(5):542–8.

491. Lekmanov AU, Erpuleva Yu V., Zolkina I V., Rossaus PA. Study of glutamine solution use efficiency in pediatric patients with heavy thermic burns and concomitant injuries in the intensive care unit. 2013;

492. Lema-Zuluaga GL, Fernandez-Laverde M, Correa-Varela AM, Zuleta-Tobón JJ. As-needed endotracheal suctioning protocol vs a routine endotracheal suctioning in Pediatric Intensive Care Unit: A randomized controlled trial. Colomb Med. 2018 Apr 1;49(2):148–53.

493. Li S, Ma Q, Yang Y, Lu J, Zhang Z, Jin M, et al. Novel Goal-Directed Hemodynamic Optimization Therapy Based on Major Vasopressor during Corrective Cardiac Surgery in Patients with Severe Pulmonary Arterial Hypertension: A Pilot Study. Heart Surg Forum. 2016 Dec 22;19(6):E297–302.

494. Lindsay CA, Barton P, Lawless S, Kitchen L, Zorka A, Garcia J, et al. Pharmacokinetics and pharmacodynamics of milrinone lactate in pediatric patients with septic shock. J Pediatr. 1998;132(2):329–34.

495. Liu JF, Xie WP, Lei YQ, Cao H, Yu XR, Chen Q. Effects of different feeding intervals on the feeding outcomes of infants who underwent surgical repair of ventricular septal defects. J Card Surg. 2021 Nov 1;36(11):4134–8.

496. Liu M, Zheng P, Zheng X ying, Meng X ka. Rational control on postoperative blood glucose levels in infants with congenital heart disease. Chinese Critical Care MEdicine. 2012;24(4):244–6.

497. Liu MH, Zhu LH, Peng JX, Zhang XP, Xiao ZH, Liu QJ, et al. Effect of personalized music intervention in mechanically ventilated children in the PICU: A pilot study. Pediatric Critical Care Medicine. 2019 Jan 1;21(1):E8–14.

498. Liu W, Zuo Z, Ma R, Zhang X. Effect of mechanical cleaning of endotracheal tubes with sterile urethral catheters to reduce Biofilm formation in ventilator patients. Pediatric Critical Care Medicine. 2013;14(7):e338–43.

499. Long EJ, Shann F, Pearson G, Buckley D, Butt W. A randomised controlled trial of plasma filtration in severe paediatric sepsis. Critical Care and Resuscitation [Internet]. 2013;15(3):198–204. Available from: www.jficm.anzca.edu.au/aaccm/journal/publi-

500. Lopez Castilla JD, Martinez Carapeto I, Fresneda Gutierrez R, Cano Franco J, Sanchez Valderrabano E, Charlo Molina T, et al. Efficacy and safety of isotonic saline serum as a maintenance therapy serum after general surgery in pediatrics patients. Acta Pediatr Esp. 2019;77(11–12):181–7.

501. Macrae D, Grieve R, Allen E, Sadique Z, Morris K, Pappachan J, et al. A Randomized Trial of Hyperglycemic Control in Pediatric Intensive Care. New England Journal of Medicine. 2014 Jan 9;370(2):107–18.

502. Mancy AS, Shaheen S, Albaghdady A, Sabri NA. Efficacy and safety of Colistin-Imipenem/Cilastatin combination Therapy for Multidrug-Resistant Gram-Negative Bacteria Infections in Critically Ill Pediatric Patients. Res J Pharm Technol. 2022 Mar 1;15(3):1059–63.

503. Marseglia L, Aversa S, Barberi I, Salpietro CD, Cusumano E, Speciale A, et al. High endogenous melatonin levels in critically ill children: A pilot study. Journal of Pediatrics. 2013 Feb;162(2):357–60.

504. Martínez Carapeto I, Domingo J, Castilla L, Fresneda Gutiérrez R. A comparison of post-surgical plasma glucose levels in patients on fluids with different glucose concentrations. An Pediatr (Barc) [Internet]. 2018;89(2):98–103. Available from: www.analesdepediatria.org

505. McKinley DF, Kinney SB, Copnell B, Shann F. Long-term effects of saline instilled during endotracheal suction in pediatric intensive care: A randomized trial. American Journal of Critical Care. 2018 Nov 1;27(6):486–94.

506. Menon K, McNally D, O’Hearn K, Acharya A, Wong HR, Lawson M, et al. A Randomized Controlled Trial of Corticosteroids in Pediatric Septic Shock: A Pilot Feasibility Study. Pediatric Critical Care Medicine. 2017 Jun 1;18(6):505–12.

507. Moler FW, Silverstein FS, Holubkov R, Slomine BS, Christensen JR, Nadkarni VM, et al. Therapeutic Hypothermia after Out-of-Hospital Cardiac Arrest in Children. New England Journal of Medicine. 2015 May 14;372(20):1898–908.

508. Moler FW, Silverstein FS, Holubkov R, Slomine BS, Christensen JR, Nadkarni VM, et al. Therapeutic Hypothermia after In-Hospital Cardiac Arrest in Children. New England Journal of Medicine. 2017 Jan 26;376(4):318–29.

509. Momeni M, Rubay J, Matta A, Rennotte MT, Veyckemans F, Poncelet AJ, et al. Levosimendan in congenital cardiac surgery: A randomized, double-blind clinical trial. J Cardiothorac Vasc Anesth. 2011 Jun;25(3):419–24.

510. Naeem M, Alem H Al, Shehri A Al, Al-Jeraisy M. Effect of N-Methyl-D-aspartate receptor antagonist dextromethorphan on opioid analgesia in pediatric intensive care unit. Pain Res Manag. 2016;2016.

511. Nair S, Kazi A. Efficacy of thoracic mobility and breathing exercises on chest expansion and pulmonary function values in post intra-cardiac repair surgery patients. Journal of Pharmaceutical Sciences and Research. 2019;11(10):3458–61.

512. Nazarchuk OA, Dmytriiev D V., Dmytriiev KD. Clinical, microbiological research of the effectiveness of inhalation use of quarternary ammonium antiseptic in the prevention and treatment of infectious respiratory complications in critically ill children. Biomedical Research and Therapy. 2018;5(12):2850–62.

513. Ning B, Ye S, Lyu Y, Yin F, Chen Z. Effect of high-volume hemofiltration on children with sepsis. Transl Pediatr. 2020 Apr 1;9(2):101–7.

514. Nomura N, Asano M, Saito T, Nakayama T, Mishima A. Sivelestat attenuates lung injury in surgery for congenital heart disease with pulmonary hypertension. Annals of Thoracic Surgery. 2013 Dec;96(6):2184–91.

515. Öz Ö, Uysal G, Düzkaya DS. Effect of Two Bathing Methods on Physiologic Parameters in Pediatric Intensive Care. Clin Nurs Res. 2022 Jun 1;31(5):858–65.

516. Peiravian F, Amirghofran AA, Borzouee M, Ajami GH, Sabri MR, Kolaee S. Oral Sildenafil to Control Pulmonary Hypertension after Congenital Heart Surgery. Asian Cardiovascular Thoracic Annals. 2007;15(2):113–7.

517. Pemberton VL, Browning B, Webster A, Dean JM, Moler FW. Therapeutic hypothermia after pediatric cardiac arrest trials: The vanguard phase experience and implications for other trials. Pediatric Critical Care Medicine. 2013 Jan;14(1):19–26.

518. Penk JS, Lefaiver CA, Brady CM, Steffensen CM, Wittmayer K. Intermittent Versus Continuous and Intermittent Medications for Pain and Sedation after Pediatric Cardiothoracic Surgery; A Randomized Controlled Trial. Crit Care Med. 2018 Jan 1;46(1):123–9.

519. Piva J, Alquati T, Garcia PC, Fiori H, Einloft P, Bruno F. Norepinephrine infusion increases urine output in children under sedative and analgesic infusion. Rev Assoc Med Bras. 2014 May 1;60(3):208–15.

520. Popov D, Yaroustovsky M, Lobacheva G. Prevention of infectious complications after heart surgery in children: Procalcitonin-guided strategy. Kardiochirurgia i Torakochirurgia Polska. 2014;11(2):140–4.

521. Portman MA, Slee A, Olson AK, Cohen G, Karl T, Tong E, et al. Triiodothyronine supplementation in infants and children undergoing cardiopulmonary bypass (TRICC): A multicenter placebo-controlled randomized trial: Age analysis. Circulation. 2010 Sep 14;122(11 SUPPL. 1).

522. Prasad SR, Simha PP, Jagadeesh AM. Comparative study between dexmedetomidine and fentanyl for sedation during mechanical ventilation in post-operative paediatric cardiac surgical patients. Indian J Anaesth. 2012;56(6):547–52.

523. Rajput RS, Das S, Makhija N, Airan B. Efficacy of dexmedetomidine for the control of junctional ectopic tachycardia after repair of tetralogy of Fallot. Ann Pediatr Cardiol. 2014 Sep 1;7(3):167–72.

524. Ramaswamy KN, Singhi S, Jayashree M, Bansal A, Nallasamy K. Double-Blind Randomized Clinical Trial Comparing Dopamine and Epinephrine in Pediatric Fluid-Refractory Hypotensive Septic Shock∗. Pediatric Critical Care Medicine. 2016 Nov 1;17(11):e502–12.

525. Ramnarayan P, Lister P, Dominguez T, Habibi P, Edmonds N, Canter RR, et al. FIRST-line support for Assistance in Breathing in Children (FIRST-ABC): A multicentre pilot randomised controlled trial of high-flow nasal cannula therapy versus continuous positive airway pressure in paediatric critical care. Crit Care. 2018 Jun 4;22(1).

526. Rennick JE, Stremler R, Horwood L, Aita M, Lavoie T, Majnemer A, et al. A pilot randomized controlled trial of an intervention to promote psychological well-being in critically ill children: Soothing through touch, reading, and music. Pediatric Critical Care Medicine. 2018 Jul 1;19(7):e358–66.

527. Ritu, Jhamb U. Dexamethasone in prevention of postextubation stridor in ventilated children: A randomized, double-blinded, placebo-controlled trial. Vol. 24, Indian Journal of Critical Care Medicine. Jaypee Brothers Medical Publishers (P) Ltd; 2020. p. 1230–5.

528. Robert SM, Borasino S, Dabal RJ, Cleveland DC, Hock KM, Alten JA. Postoperative hydrocortisone infusion reduces the prevalence of low cardiac output syndrome after neonatal cardiopulmonary bypass. Pediatric Critical Care Medicine. 2015 Sep 1;16(7):629–36.

529. Rodríguez-Moya VS, Del M, Machado-Lubián C, Barrese-Pérez Y, Ávila-Albuerne Y, Uranga-Piña R, et al. Cuban Exogenous Pulmonary Surfactant in Treatment of Pediatric Acute Respiratory Distress Syndrome. MEDICC Rev. 2017;19(3):24–31.

530. Roshanzamiri S, Alemzadeh M, Ahmadizadeh SN, Behzad A, Hashemi SM, Salamzadeh J, et al. Probiotic prophylaxis to prevent ventilator-associated pneumonia in children on mechanical ventilation: A randomized double-blind clinical trial. Front Pediatr. 2022 Nov 15;10.

531. Ryerson LM, Mackie AS, Atallah J, Joffe AR, Rebeyka IM, Ross DB, et al. Prophylactic peritoneal dialysis catheter does not decrease time to achieve a negative fluid balance after the Norwood procedure: A randomized controlled trial. Journal of Thoracic and Cardiovascular Surgery. 2015;149(1):222–8.

532. Sachdev A, Punia S, Gupta D, Gupta N, Gupta S. Non-invasive positive pressure ventilation immediately after extubation in children - a randomized study. Journal of Pediatric Critical Care. 2019;6(6):15.

533. Sadek El Derh M, Mohamed Abdelaziz N, Abdel Twab SM. High Dose Methylprednisolone versus Low Dose in Correction of Congenital Acyanotic Heart Disease. Egypt J Anaesth. 2022;38(1):220–8.

534. Salamah A, Mehrez M, Faheem A, Doaa &, Amrousy E. Efficacy of Citicoline as a Neuroprotector in children with post cardiac arrest: a randomized controlled clinical trial. Eur J Pediatr [Internet]. 2021;180:1249–55. Available from: www.pactr.samrc.ac.za

535. Salarian S, Mirrahimi B, Bagheri B. The Rate of Self-Extubation in Pediatric Intensive Care Unit Following Administration of Fentanyl, Midazolam and Midazolam-Fentanyl Combination: A Comparative Study. Int J Pediatr [Internet]. 2018;6(49). Available from: http://ijp.mums.ac.ir

536. Salarian S, Khosravi R, Khanbabaei G, Bagheri B. Impact of oral clonidine on duration of opioid and benzodiazepine use in mechanically ventilated children: A randomized, double-blind, placebo-controlled study. Iranian Journal of Pharmaceutical Research. 2019 Sep 1;18(4):2157–62.

537. Saleh A, Hassan P. The Effect of Pre-emptive Oral Melatonin versus Placebo on Post-operative Analgesia in Infants after Thoracotomy for Closed Cardiac Surgeries: A Randomized Controlled Study. Open Access Maced J Med Sci. 2022 Sep 7;10(B):2195–201.

538. Saleh RH. Randomized controlled comparative trial between low dose dexmedetomidine sedation and that of fentanyl in children after surgical procedures in surgical Pediatric Intensive Care Unit. Egypt J Anaesth. 2016 Jan 1;32(1):137–42.

539. Samransamruajkit R, Rassameehirun C, Pongsanon K, Huntrakul S, Deerojanawong J, Sritippayawan S. A comparison of clinical efficacy between high frequency oscillatory ventilation and conventional ventilation with lung volume recruitment in pediatric acute respiratory distress syndrome: A randomized controlled trial. From: Indian Journal of Critical Care Medicine. 2016;20(2).

540. Schults JA, Cooke M, Long D, Schibler A, Ware RS, Charles K, et al. Normal saline and lung recruitment with paediatric endotracheal suction (NARES): A pilot, factorial, randomised controlled trial. Australian Critical Care. 2021 Nov 1;34(6):530–8.

541. Shah H, Bhavsar M, Pandya M. Comparative study of levosimendan versus milrinone for paediatric cardiac surgery patients operated with cardio-pulmonary bypass. Int J Res Med. 2013;2(4).

542. Shaikh F, Janaapureddy YR, Mohanty S, Reddy PK, Sachane K, Dekate PS, et al. Utility of endotracheal tube cuff pressure monitoring in mechanically ventilated (MV) children in preventing post-extubation stridor (PES). Indian Journal of Critical Care Medicine. 2021;25(2):181–4.

543. Sharma VK, Joshi S, Joshi A, Kumar G, Arora H, Garg A. Does intravenous sildenafil clinically ameliorate pulmonary hypertension during perioperative management of congenital heart diseases in children?-A prospective randomized study. Ann Card Anaesth. 2015 Oct 1;18(4):510–6.

544. Shime N, Kato Y, Kosaka T, Kokufu T, Yamagishi M, Fujita N. Glycopeptide pharmacokinetics in current paediatric cardiac surgery practice. European Journal of Cardio-thoracic Surgery. 2007 Oct;32(4):577–81.

545. Siddiqu NUR, Merchant Q, Hasan BS, Rizvi A, Amanullah M, Rehmat A, et al. Comparison of enteral versus intravenous potassium supplementation in hypokalaemia in paediatric patients in intensive care post cardiac surgery: Open-label randomised equivalence trial (EIPS). BMJ Open. 2017 May 1;7(5).

546. Sitthikarnkha P, Samransamruajkit R, Prapphal N, Deerojanawong J, Sritippayawan S. High-flow nasal cannula versus conventional oxygen therapy in children with respiratory distress. Indian Journal of Critical Care Medicine. 2018;22(5).

547. Soliman R, Saad D. Assessment the effect of dexmedetomidine on incidence of paradoxical hypertension after surgical repair of aortic coarctation in pediatric patients. Ann Card Anaesth. 2018 Jan 1;21(1):26–33.

548. Spinella PC, Tucci M, Fergusson DA, Lacroix J, Hébert PC, Leteurtre S, et al. Effect of Fresh vs Standard-issue Red Blood Cell Transfusions on Multiple Organ Dysfunction Syndrome in Critically Ill Pediatric Patients: A Randomized Clinical Trial. JAMA - Journal of the American Medical Association. 2019 Dec 10;322(22):2179–90.

549. Staveski SL, Boulanger K, Erman L, Lin L, Almgren C, Journel C, et al. The impact of massage and reading on children’s pain and anxiety after cardiovascular surgery: A pilot study. Pediatric Critical Care Medicine. 2018;19(8):725–32.

550. Talwar S, Selvam MS, Makhija N, Lakshmy R, Choudhary SK, Sreenivas V, et al. Effect of administration of allopurinol on postoperative outcomes in patients undergoing intracardiac repair of tetralogy of Fallot. Journal of Thoracic and Cardiovascular Surgery. 2018 Jan 1;155(1):335–43.

551. Talwar S, Harshavardhan N, Kapoor PM, Makhija N, Rajashekar P, Sreenivas V, et al. Plasmalyte-A Based del Nido Cardioplegia Versus Plain Ringer Based del Nido Cardioplegia: Double-Blind Randomized Trial. World J Pediatr Congenit Heart Surg. 2022 Mar 1;13(2):187–95.

552. Tang M, Feng M, Chen L, Zhang J, Ji P, Luo S. Closed blood conservation device for reducing catheter-related infections in children after cardiac surgery. Crit Care Nurse. 2014;34(5):53–60.

553. Tehrani RB, Farzin AO, Fani K, Heidarpour A. The Effect of Oral Triiodothyronine in Outcome of Pediatric Congenital Cardiac Surgery. Journal of Cellular & Molecular Anesthesia (JCMA). 2020;5(3):150–6.

554. Tejiram S, Sen S, Romanowski KS, Greenhalgh DG, Palmieri TL. Examining 1:1 vs. 4:1 Packed Red Blood Cell to Fresh Frozen Plasma Ratio Transfusion during Pediatric Burn Excision. Journal of Burn Care and Research. 2020;41(3):443–9.

555. Testa G, Iodice F, Ricci Z, Vitale V, De Razza F, Haiberger R, et al. Comparative evaluation of high-flow nasal cannula and conventional oxygen therapy in paediatric cardiac surgical patients: A randomized controlled trial. Interact Cardiovasc Thorac Surg. 2014;19(3):456–61.

556. Thorlacius EM, Suominen PK, Wåhlander H, Keski-Nisula J, Vistnes M, Ricksten SE, et al. The Effect of Levosimendan Versus Milrinone on the Occurrence Rate of Acute Kidney Injury Following Congenital Heart Surgery in Infants: A Randomized Clinical Trial. Pediatric Critical Care Medicine. 2019 Oct 1;20(10):947–56.

557. Tiacharoen D, Lertbunrian R, Veawpanich J, Suppalarkbunlue N, Anantasit N. Protocolized sedative weaning vs usual care in pediatric critically ill patients: A pilot randomized controlled trial. Indian Journal of Critical Care Medicine. 2020;24(6):451–8.

558. Tirotta CF, Munro HM, Salvaggio J, Madril D, Felix DE, Rusinowski L, et al. Continuous incisional infusion of local anesthetic in pediatric patients following open heart surgery. Paediatr Anaesth. 2009 Jun;19(6):571–6.

559. Udurgucu M, Albayrak H, Kinik Kaya HE, Yener N. Comparison of Two Weaning Methods from Heated Humidified High-Flow Nasal Cannula Therapy in Pediatric Intensive Care Unit. Pediatr Allergy Immunol Pulmonol. 2022 Jun 1;35(2):79–85.

560. Verdesoto Rodriguez MC, Spenceley N, Ilina M, Danton MHD. A Prospective Randomized Blinded Trial of Remote Ischemic Preconditioning in Children Undergoing Cardiac Surgery. Semin Thorac Cardiovasc Surg. 2020 Jun 1;32(2):313–22.

561. Verlaat CWM, Heesen GP, Vet NJ, De Hoog M, Van Der Hoeven JG, Kox M, et al. Randomized controlled trial of daily interruption of sedatives in critically ill children. Paediatr Anaesth. 2013 Feb;24(2):151–6.

562. Vet NJ, de Wildt SN, Verlaat CWM, Knibbe CAJ, Mooij MG, van Woensel JBM, et al. A randomized controlled trial of daily sedation interruption in critically ill children. Intensive Care Med. 2016 Feb 1;42(2):233–44.

563. Wang W, Qi J, Lu H, Wang Y, Li Y, Zhou R, et al. Fast track radical surgery in pediatric patients with congenital heart diseases [Internet]. Vol. 12, Int J Clin Exp Med. 2019. Available from: www.ijcem.com/

564. Wang Y, Gao L, Yang Z, Chen F, Zhang Y. Effects of probiotics on ghrelin and lungs in children with acute lung injury: A double-blind randomized, controlled trial. Pediatr Pulmonol. 2018 Feb 1;53(2):197–203.

565. Wang Y, Yang Z, Gao L, Cao Z, Wang Q. Effects of a single dose of vitamin D in septic children: a randomized, double-blinded, controlled trial. Journal of International Medical Research. 2020 Jun 1;48(6).

566. Wang Y, Liu YZ, Chen YZ, Wu W, Hong XY, Gao ML. Effect of cyclic management strategy with rScO2 and Pcv-aCO2 as the main goal on the short term prognosis of children with cyanotic congenital heart disease. Medical Journal of Chinese People’s Liberation Army. 2022 Jul 28;47(7):709–16.

567. Welzing L, Oberthuer A, Junghaenel S, Harnischmacher U, Stützer H, Roth B. Remifentanil/midazolam versus fentanyl/midazolam for analgesia and sedation of mechanically ventilated neonates and young infants: A randomized controlled trial. Intensive Care Med. 2012 Jun;38(6):1017–24.

568. Wijakprasert P, Chomchoey J. High-Flow Nasal Cannula versus Conventional Oxygen Therapy in Post-Extubation Pediatric Patients: A Randomized Controlled Trial. J Med Assoc Thai. 2018;101(10):1331–6.

569. Willems A, De Groote F, Schmartz D, Fils JF, Van Der Linden P. Does a balanced colloid decrease perioperative blood loss in paediatric cardiac surgery: A double-blinded randomized controlled trial? Eur J Anaesthesiol. 2021 Sep 1;38(9):923–31.

570. Willson DF, Thomas NJ, Tamburro R, Truemper E, Truwit J, Conaway M, et al. Pediatric calfactant in acute respiratory distress syndrome trial. Pediatric Critical Care Medicine. 2013;14(7):657–65.

571. Wolf A, McKay A, Spowart C, Granville H, Boland A, Petrou S, et al. Prospective multicentre randomised, double-blind, equivalence study comparing clonidine and midazolam as intravenous sedative agents in critically ill children: the SLEEPS (Safety profile, Efficacy and Equivalence in Paediatric intensive care Sedation) study. Health Technol Assess (Rockv). 2014;18(71):1–242.

572. Xie J, Zhu L, Zhu T, Jian Y, Ding Y, Zhou M, et al. Zinc supplementation reduces Candida infections in pediatric intensive care unit: a randomized placeboocontrolled clinical trial. J Clin Biochem Nutr |. 2018;1–4.

573. Xie J, Zhu L, Zhu T, Jian Y, Ding Y, Zhou M, et al. Vitamin D-supplemented yogurt drink reduces Candida infections in a paediatric intensive care unit: a randomised, placebo-controlled clinical trial. Journal of Human Nutrition and Dietetics. 2019 Aug 1;32(4):512–7.

574. Xu N, Qiao L, Yin L, Li H. Value of pleth variability index to guide fluid resuscitation in children with sepsis. Biomedical Research [Internet]. 2017;28(22):9875–8. Available from: www.biomedres.info

575. Yu XR, Huang ST, Xu N, Wang LW, Wang ZC, Cao H, et al. The effect of early oral stimulation with breast milk on the feeding behavior of infants after congenital cardiac surgery. J Cardiothorac Surg. 2020 Oct 9;15(1).

576. Yu XR, Huang ST, Xu N, Dai WS, Wang ZC, Cao H, et al. Comparison of the Effect of Breast Milk and Sodium Bicarbonate Solution for Oral Care in Infants with Tracheal Intubation after Cardiothoracic Surgery. Breastfeeding Medicine. 2021 Jul 1;16(7):568–72.

577. Yu XR, Xu N, Huang ST, Zhang QL, Wang ZC, Cao H, et al. Effects of different oral care strategies on postoperative pneumonia in infants with mechanical ventilation after cardiac surgery: A prospective randomized controlled study. Transl Pediatr. 2021 Feb 1;10(2):359–65.

578. Yu XR, Lei YQ, Liu JF, Wang ZC, Cao H, Chen Q. Effect of breast milk oral care in infants who underwent surgical correction of ventricular septal defect. Cardiol Young. 2021 Dec 22;31(12):2015–8.

579. Yuan YH, Xiao ZH, Zhang H, Fan JH, Zhang XP, Lu XL, et al. Impact of continuous blood purification on T cell subsets in children with severe sepsis. Chinese Journal of Contemporary Pediatrics. 2014 Feb 15;16(2):194–7.

580. Yuan YH, Zhang H, Xiao ZH, Zhang XP, Lu XL, Xu ZY, et al. Efficacy of plasma exchange in children with severe hemophagocytic syndrome: a prospective randomized controlled trial. Chinese Journal of Contemporary Pediatrics. 2022 Mar 1;24(3):249–54.

581. Yung M, Letton G, Keeley S. Controlled trial of Hartmann’s solution versus 0.9% saline for diabetic ketoacidosis. J Paediatr Child Health. 2017 Jan 1;53(1):12–7.

582. Zhang Y, Chen S, Gong H, Zhan B. Efficacy of Bilateral Transversus Thoracis Muscle Plane Block in Pediatric Patients Undergoing Open Cardiac Surgery. J Cardiothorac Vasc Anesth. 2020 Sep 1;34(9):2430–4.

583. Zhang Y, Min J, Chen S. Perioperative Pain Management With Bilateral Pecto-intercostal Fascial Block in Pediatric Patients Undergoing Open Cardiac Surgery. Front Cardiovasc Med. 2022 Jun 22;9.

584. Zheng YR, Chen YK, Lin SH, Cao H, Chen Q. Effect of High-Frequency Oscillatory Ventilation, Combined With Prone Positioning, in Infants With Acute Respiratory Distress Syndrome After Congenital Heart Surgery: A Prospective Randomized Controlled Trial. J Cardiothorac Vasc Anesth. 2022 Oct 1;36(10):3847–54.

585. Zhou J, Lv Y, Hu Z, Qiu Z, Peng R, Xue Y. Sildenafil in the treatment of postoperative hypoxemia with high resistance in cyanotic congenital heart disease. Am J Biochem Biotechnol. 2017 Jan 1;13(3):140–7.
