# Supplementary material for: Reporting of social determinants of health in randomized controlled trials conducted in the pediatric intensive care unit
Source: Front Pediatr. 2024 Feb 1;12:1329648. doi: 10.3389/fped.2024.1329648 (PMC10867174; doi:10.3389/fped.2024.1329648)
Supplement: Supplementary file 2 [file Table2.docx]

Supplementary file 2: Table of in text citations

| **RCT Description** | **Number of RCTs** | **Reference numbers from Supplementary file 1** |
| --- | --- | --- |
| All unique RCTs (Adelson had two studies in one paper) | 586 | 1-585 |
| Food insecurity factor reported by arm | 97 | 4–14,39,42,50,54,63, 67–69,71–148 |
| Food insecurity factor reported by cohort | 3 | 1-3 |
| Feeding/nutrition studies that also reported baseline nutritional assessments | 25 | 42,67-90 |
| Mechanical ventilation studies that also reported baseline nutritional assessments | 11 | 4-14 |
| Race/ethnicity of patient or caregiver | 73 | 1,12,16–24,27,29,30,39,43, 50,63,64,85,95,99,105,128, 149,151–153,170,174–204 |
| Race/ethnicity of patient or caregiver and was conducted in the United Statues | 61 | 1,12,16–24,27,29,30,39,43, 50, 63, 64,85,95,99,105,128, 149,151–153,170,174–204 |
| Race/ethnicity collection method: self-report | 7 | 15-21 |
| Race/ethnicity collection method: medical records | 5 | 22-26 |
| Race/ethnicity collection method: study team | 3 | 27-29 |
| Race/ethnicity collection method: family | 1 | 30 |
| Studies that investigated affordable healthcare services factors | 10 | 31-40 |
| Patient travel distance of health services by cohort | 4 | 31,32,35,37 |
| Patient travel distance of health services by arm | 1 | 32 |
| Presence of PICU at nearest hospital | 1 | 32 |
| Health insurance status | 1 | 35 |
| Immunization status | 1 | 34 |
| Urban vs rural reported by cohort | 4 | 36, 38-40 |
| Urban vs rural reported by study arm | 1 | 33 |
| Socioeconomic status of patient/family | 10 | 15–19,37,38,41–43 |
| Socioeconomic status by cohort | 6 | 16,37,38,41-43 |
| Socioeconomic status by study arm | 4 | 15,17-19 |
| Immediate catchment area | 1 | 41 |
| Recruited patient cohort | 1 | 43 |
| Marital status by study arm | 4 | 15,17-19 |
| Marital status by cohort | 2 | 16,35 |
| Household composition by study arm | 1 | 21 |
| Household composition by cohort | 1 | 36 |
| Caregiver smoking by study arm | 7 | 43-49 |
| Caregiver smoking by cohort | 1 | 50 |
| High altitude | 2 | 51,52 |
| Organophosphate poisoning | 1 | 53 |
| Severe asthma studies | 16 | 20,39,43,45,49,149–159 |
| Severe asthma studies that reported on smoking at home | 3 | 43,45,49 |
| Bronchiolitis studies | 25 | 1,12,16-24,27,29,30,39,43, 50, 63,64,85,95,99,105,128, 149,151–153,170,174–204 |
| Bronchiolitis studies that reported on smoking at home | 3 | 44,47,50 |
| Education of caregiver by study arm | 4 | 15,17-19 |
| Education of caregiver by cohort | 2 | 16,35 |
| Employment status by study arm | 1 | 18 |
| Employment status by cohort | 1 | 35 |
| Explicitly stated that study included patients over 18 years of age | 15 | 18,22,39,43,54-64 |
| Child protected services involvement | 2 | 65,66 |
