# Supplementary material for: Reporting of social determinants of health in randomized controlled trials conducted in the pediatric intensive care unit
Source: Front Pediatr. 2024 Feb 1;12:1329648. doi: 10.3389/fped.2024.1329648 (PMC10867174; doi:10.3389/fped.2024.1329648)
Supplement: Supplementary file 3 [file Table3.docx]

Supplementary file 3: Number of patients randomized per country.

| **Country for single centre studies** | **Patients randomized, n (%)** |
| --- | --- |
| United States | 27185 (27.9) |
| United Kingdom | 24120 (24.8) |
| India | 5893 (6.1) |
| China | 5166 (5.3) |
| Australia | 2611 (2.7) |
| Brazil | 2539 (2.6) |
| Turkey | 2277 (2.3) |
| Egypt | 2140 (2.2) |
| Germany | 1810 (1.9) |
| Iran | 1664 (1.7) |
| Spain | 1596 (1.6) |
| The Netherlands | 1187 (1.2) |
| Thailand | 1032 (1.1) |
| Canada | 906 (0.9) |
| Belgium | 862 (0.9) |
| Italy | 566 (0.6) |
| South Korea | 507 (0.5) |
| Japan | 502 (0.5) |
| Argentina | 480 (0.5) |
| France | 467 (0.5) |
| Israel | 357 (0.4) |
| Indonesia | 338 (0.3) |
| Portugal | 268 (0.3) |
| Tunisia | 268 (0.3) |
| Chile | 264 (0.3) |
| Bangladesh | 255 (0.3) |
| Finland | 202 (0.2) |
| Pakistan | 197 (0.2) |
| Vietnam | 196 (0.2) |
| Greece | 187 (0.2) |
| Russia | 143 (0.1) |
| South Africa | 129 (0.1) |
| Saudi Arabia | 122 (0.1) |
| Switzerland | 117 (0.1) |
| Austria | 102 (0.1) |
| Colombia | 92 (0.1) |
| Cuba | 73 (0.1) |
| Taiwan | 60 (0.1) |
| Croatia | 46 (0) |
| Poland | 41 (0) |
| Malaysia | 30 (0) |
| Ukraine | 30 (0) |
| Philippines | 27 (0) |
| **Countries for multi-centre trials** |  |
| Canada, France, Italy, Israel, United States | 1538 (1.6) |
| Belgium, Canada, The Netherlands | 1440 (1.5) |
| Australia, New Zealand, The Netherlands | 1371 (1.4) |
| Canada, United States | 803 (0.8) |
| Australia, United States | 713 (0.7) |
| Belgium, Canada, United Kingdom, United States | 648 (0.7) |
| Australia, Chile, France, Germany, Italy, Mexico, Poland, Slovakia, Switzerland, United Kingdom | 496 (0.5) |
| United Kingdom, United States | 395 (0.4) |
| Brazil, China, Russia | 394 (0.4) |
| Canada, United Kingdom, United States | 329 (0.3) |
| Australia, New Zealand | 324 (0.3) |
| France, Norway, Spain, The Netherlands, United Kingdom | 269 (0.3) |
| Argentina, Spain | 257 (0.3) |
| Canada, France, United Kingdom | 225 (0.2) |
| Chile, United States | 165 (0.2) |
| Belgium, Italy, The Netherlands, United Kingdom | 145 (0.1) |
| Egypt, Saudi Arabia | 116 (0.1) |
| Australia, Canada, Israel, New Zealand, South Korea, United States | 110 (0.1) |
| Australia, New Zealand, United States | 77 (0.1) |
| France, Switzerland | 73 (0.1) |
| Finland, Sweden | 72 (0.1) |
| Argentina, Brazil, Canada, Chile, Peru, South Africa, United States | 61 (0.1) |
| Australia, Canada, New Zealand | 55 (0.1) |
| Australia, New Zealand, United Kingdom | 48 (0) |
| Canada, France | 40 (0) |
| The Netherlands, United Kingdom | 40 (0) |
| Austria, Germany | 38 (0) |
| Brazil, United Kingdom | 30 (0) |
| France, United States | 18 (0) |
